# Temporal tracking of microglial and monocyte single-cell transcriptomics in lethal flavivirus infection

**DOI:** 10.1186/s40478-023-01547-4

**Published:** 2023-04-04

**Authors:** Alanna G. Spiteri, Claire L. Wishart, Duan Ni, Barney Viengkhou, Laurence Macia, Markus J. Hofer, Nicholas J. C. King

**Affiliations:** 1 Viral Immunopathology Laboratory, Infection, Immunity and Inflammation Research Theme, School of Medical Sciences, Faculty of Medicine and Health, The University of Sydney, Sydney, NSW 2006 Australia; 2 Sydney Cytometry, The University of Sydney and Centenary Institute, Sydney, NSW 2006 Australia; 3 Ramaciotti Facility for Human Systems Biology, The University of Sydney and Centenary Institute, Sydney, NSW 2006 Australia; 4 Charles Perkins Centre, The University of Sydney, Sydney, NSW 2006 Australia; 5 Chronic Diseases Research Theme, School of Medical Sciences, Faculty of Medicine and Health, The University of Sydney, Sydney, NSW 2006 Australia; 6 School of Life and Environmental Sciences, The University of Sydney, Sydney, NSW 2006 Australia; 7 The University of Sydney Institute for Infectious Diseases, The University of Sydney, Sydney, NSW 2006 Australia; 8 Sydney Nano, The University of Sydney, Sydney, NSW 2006 Australia

**Keywords:** Microglia, Monocyte-derived cells, Neuroinflammation, Virus-induced encephalitis, Microglia depletion, CNS infection

## Abstract

As the resident parenchymal myeloid population in the central nervous system (CNS), microglia are strategically positioned to respond to neurotropic virus invasion and have been implicated in promoting both disease resolution and progression in the acute and post-infectious phase of virus encephalitis. In a mouse model of West Nile virus encephalitis (WNE), infection of the CNS results in recruitment of large numbers of peripheral immune cells into the brain, the majority being nitric oxide (NO)-producing Ly6C^hi^ inflammatory monocyte-derived cells (MCs). In this model, these cells enhance immunopathology and mortality. However, the contribution of microglia to this response is currently undefined. Here we used a combination of experimental tools, including single-cell RNA sequencing (scRNA-seq), microglia and MC depletion reagents, high-dimensional spectral cytometry and computational algorithms to dissect the differential contribution of microglia and MCs to the anti-viral immune response in severe neuroinflammation seen in WNE. Intriguingly, analysis of scRNA-seq data revealed 6 unique microglia and 3 unique MC clusters that were predominantly timepoint-specific, demonstrating substantial transcriptional adaptation with disease progression over the course of WNE. While microglia and MC adopted unique gene expression profiles, gene ontology enrichment analysis, coupled with microglia and MC depletion studies, demonstrated a role for both of these cells in the trafficking of peripheral immune cells into the CNS, T cell responses and viral clearance. Over the course of infection, microglia transitioned from a homeostatic to an *anti-viral* and then into an *immune cell-recruiting* phenotype. Conversely, MC adopted *antigen-presenting*, *immune cell-recruiting* and *NO-producing* phenotypes, which all had anti-viral function. Overall, this study defines for the first time the single-cell transcriptomic responses of microglia and MCs over the course of WNE, demonstrating both protective and pathological roles of these cells that could potentially be targeted for differential therapeutic intervention to dampen immune-mediated pathology, while maintaining viral clearance functions.

**Supplementary Information:**

The online version contains supplementary material available at 10.1186/s40478-023-01547-4.

## Introduction

Microglia, the resident parenchymal myeloid cells of the central nervous system (CNS), are thought to coordinate important anti-viral responses during CNS infection. Neurotropic virus infection leading to encephalitis, meningitis, or flaccid paralysis, if not fatal, can cause severe cognitive, learning and memory deficits that often worsen over time [21, 48, 54, 64, 98, 102]. Following its emergence in the Americas at the turn of this century, West Nile virus (WNV), a mosquito-borne, neurotropic flavivirus, now has the largest world-wide distribution among *Flaviviridae* [2, 14, 40, 62, 91]. However, as yet there are no vaccines licensed for use in humans and treatment relies largely on palliative support, with anti-viral strategies of limited benefit [14, 90]. Understanding disease pathogenesis is thus required to devise effective immunomodulatory treatment strategies in viral encephalitis. Microglia have been implicated in promoting both disease resolution and progression in the acute and post-infectious phase of encephalitis, respectively [19, 21, 70, 97]. Although these cells represent potentially useful therapeutic targets, their precise temporal and functional contribution to coordinating an anti-viral response is incompletely understood.

Studying the role of microglia in health and disease has until recently been extremely difficult. Firstly, culturing primary adult microglia is hampered by the limited number of cells that can be isolated from the CNS and the difficulty in maintaining their viability [4]. Secondly, studies showing the importance of brain-specific signals and environmental cues in maintaining a functional microglial signature in the intact brain [9, 29] have shifted the emphasis from in vitro to in vivo studies. Recently, the development of microglia-specific markers, such as TMEM119 [3] and P2RY12 [9], and microglia-depleting drugs such as the CSF-1R inhibitor, PLX5622 [73], have helped to resolve more clearly the identity and function of this population. However, these tools have proved less reliable in vivo during severe neuroinflammation. This is because microglia-specific markers are downregulated by microglia and/or expressed de novo by infiltrating monocyte-derived cells (MCs) from the bone marrow (BM), confounding accurate discrimination between these two cell types [16, 31, 44, 76, 77, 96]. Furthermore, PLX5622 targets both microglia and peripheral cell subsets, making it difficult to disambiguate functions in microglia and infiltrating monocytes [41, 42, 75].

The evolution of tools to study microglia has nevertheless re-shaped our understanding of microglial responses to CNS pathogen evasion. Prior to the availability of ‘microglia-specific’ reagents, studies investigating viral encephalitis relied on cell line cultures, inaccurate identification of microglia and ex vivo slice culture models. These studies suggested that microglia were responsible for producing an array of pro-inflammatory cytokines in the CNS that promoted damage and neuroinflammation [12, 47, 50, 51, 60, 85, 100]. Intriguingly, however, the use of PLX5622, thought originally to deplete microglia specifically, showed the opposite in viral models in vivo*.* In these studies (extensively reviewed in [78]), microglia were neuroprotective following WNV [19, 70], Theiler's murine encephalomyelitis virus (TMEV) [66, 67, 99], Pseudorabies (PRV) [18], JHM strain of mouse hepatitis virus [7, 49, 68, 103], Herpes Simplex virus (HSV) [88, 92], Zika virus [17], Vesicular Stomatitis Virus (VSV) [53] and Japanese encephalitis virus [70] infection, since microglia depletion enhanced viral load and/or mortality and/or morbidity. These studies suggested that microglia perform various anti-viral functions, including phagocytosing infected neurons to prevent the fatal spread of virus in the CNS [17, 18], promoting T cell activation [7, 19, 49, 53, 67, 99, 103] and BM-derived monocyte infiltration and maturation in the CNS [18, 19, 103]. Microglia were also required to promote remyelination in the recovery phase of viral infection [49, 68].

However, many of these studies lack confirmatory evidence and do not take into account the non-microglial effects produced by depletion agents [75, 76]. Despite limited data demonstrating this [30], it was suggested that PLX5622 causes off-target effects by impairing functions of peripheral lymphoid and myeloid cells [41]. More recently, we have shown that PLX5622 targets peripheral cells in the BM, depleting and inhibiting the proliferation of mature Ly6C^hi^ monocytes and dendritic cell subsets in non-infected and WNV-infected mice. This places an important caveat on previous interpretations of data where PLX5622 was assumed to be microglia-specific [74].

In this recent work, we showed these off-target effects of PLX5622 on the BM serendipitously reduced the massive recruitment of Ly6C^hi^ inflammatory MCs into the WNV-infected CNS by 70% [75]. Inoculated intranasally, WNV enters the CNS likely via retrograde transport from peripheral neurons in the olfactory epithelium, subsequently infecting neurons in the olfactory bulbs and the posterior regions of the brain with disease progression [37, 106]. This results in the massive recruitment of Ly6C^hi^ inflammatory MCs into the parenchyma of the CNS from the BM driving severe immunopathology, neuroinflammation and lethal encephalitis [22–25, 35, 37, 84]. Thus, in this model the 70% reduction in inflammatory MCs into the CNS with PLX5622 treatment and microglia depletion ameliorated the disease phenotype and reduced neuroinflammation, despite an increase in viral load [75]. This suggests that in contrast to previously published articles [7, 19, 49, 53, 67, 99, 103], microglia are pathogenic in this model of WNV-infection and/or PLX5622 is protective in monocyte-mediated diseases by inhibiting monocyte proliferation and CNS infiltration [74].

To investigate this in detail and more clearly determine the role of microglia and MCs in WNV-infection, we used a combination of tools, including single-cell RNAsequencing (scRNA-seq), differential microglia and MC depletion reagents, high-dimensional spectral cytometry and computational analysis algorithms. Analysis of transcriptomic data showed that microglia and MCs adopt unique expression profiles that change substantially with the course of disease. We show that while microglia and MCs are responsible for the recruitment of NK and T cells into the CNS, microglia were required for CD4^+^ T cell IFN-γ production and MCs were required for CD8^+^ T cell proliferation. We further demonstrate a role for these cells in viral clearance, via the expression of anti-viral interferon-stimulated genes (ISG), microglia-mediated phagocytosis of virus-infected neurons, and potentially via the cross-presentation of antigen by microglia and MCs to CD8^+^ T cells. Together, this work demonstrates protective and pathological processes orchestrated by microglia and MC in severe neuroinflammation and CNS infection, highlighting potential therapeutic targets for immune intervention.

## Materials and methods

### WNV infection

Female 9–10-week-old C57BL/6 mice from the Animal Resource Centre (ARC) (Western Australia, Australia) were anesthetised with isoflurane prior to being infected intranasally with WNV, delivered in 10 μL of sterile PBS as previously described [25]). Mice were infected with 1.2 × 10^5^ plaque forming units (PFU), lethal in 100% of these mice. WNV Sarafend is a lab-adapted, lineage II strain, originally sourced from the John Curtin School of Medical Research and propagated in neonatal mouse brains, then used to infect confluent monolayers of vero cells at a multiplicity of infection of 5 pfu/cell [22, 25]. Infected cells were incubated for 40 h prior to freeze-thawing and clarifying the virus-rich supernatant by centrifugation. Aliquots were stored at -80 °C until use. A standard agarose virus plaque assay with baby hamster kidney cells was used to enumerate viral plaques and determine virus titers. All mouse experiments were approved by the University of Sydney Animal Ethics Committee (approval number 2019/1696) in accordance with National Health and Medical Research Council’s ethical guidelines.

### Single-cell RNA sequencing and analysis

Microglia and CNS-infiltrating MCs from mock- and/or WNV-infected mice at 5 and 7 days post infection (dpi) were collected and processed. See *Tissue processing and spectral cytometry* for a description of our brain processing procedure. Two brains were pooled per sample to obtain a sufficient number of sorted cells. Single cell suspensions were incubated with anti-CD16/32 and Zombie UV (Biolegend) viability dye, before being incubated with fluorescently-labelled antibodies. Cells were washed twice, filtered and sorted on the 10-laser Influx cell sorter using the FACSDiva Programme (BD Biosciences). The gating strategy used to identify microglia and MCs during cell sorting is shown in Additional file 1. Cells were sorted into FBS and kept on ice until they were barcoded with the single-cell mouse multiplexing kit (BD Biosciences) which uses an anti-CD45 antibody. The BD Immune response panel and 67 custom genes (total of 464 genes, Additional file 2) were sequenced on the Illumina NextSeq 1000 (300 cycles using a P1 cycle kit) with a 20% phix spike-in. Sample tag and mRNA libraries were pooled at a ratio of 1:12.5 and loaded at 650 pM, to obtain ~ 8000 reads per cell. FASTQ files were generated in BaseSpace (Illumina) and imported in SevenBridges. Sequencing reads were processed in SevenBridges using the BD Rhapsody Targeted analysis pipeline. Distribution-based error correction (DBEC) molecules per cell (DBEC_MolsPerCell) were imported into Seurat [8, 33, 69, 81] for downstream analysis. Cells in each dataset were included if the number of unique genes detected per cell were between ± 3 median absolute deviations from the median number of unique genes expressed per cell. Normalisation and variance stabilization of counts were performed using the *sctransform()* prior to running principal component analysis (PCA) and Uniform Manifold Approximation and Projection (UMAP) dimensionality reduction in Seurat (dims = 9, resolution = 0.5). The *FindNeighbours()* and *FindClusters()* functions with default parameters were then used to perform graph-based clustering on a shared nearest-neighbour graph. Differentially expressed genes (DEGs) (or top markers per cluster) were identified using the Seurat function *FindAllMarkers*(), defined as positively enriched genes with an adjusted *p* < 0.01 and log_2_FC > 0.25 (Wilcox sum rank test).

Functional gene ontology (GO) enrichment analysis from DEGs of each cluster was performed using the VISEAGO (v1.4.0) and topGO (v2.42.0) packages in R. GO biological process term enrichment was performed using the VISEAGO *create_topGOdata*() relative to the background gene expression, which was defined as the full list of genes in the Rhapsody data set. Enrichment tests were performed with Fisher’s exact test using the “elim” algorithm. Enriched GO terms were defined as terms with a minimum of ten genes mapping to a term and an adjusted p-value greater than 0.01. For visualization of GO terms, GO terms for each population were combined into a single matrix using the ViSEAGO function *build_GO_SS*() [6] and annotated using the Bioconductor *org.Mm.eg.db* database package for the mouse species [11]. UpSet visualization was performed on the significantly enriched GO term matrices using the UpSetR package (v1.4.0) [15]. Dot plot visualisation of GO terms was performed using the ggplots2 package (v3.3.3) [104, 105].

Trajectory-based analysis on scRNA-seq Rhapsody data was performed with default settings in R using Monocle3 [10, 58, 86] or Slingshot [80] on microglia and MC clusters or just microglia clusters, respectively, to determine temporal relationships between gene expression profiles. We used MG1 and MC1 or just MG1 as the starting cells (or “roots”) for Monocle3 and Slingshot, respectively.

### 2.1 Animal treatments

#### PLX5622-mediated microglia depletion

Microglia depletion was achieved with PLX5622 (Plexxikon Inc., USA) which was formulated in AIN-76A standard chow by Research diets (USA) (1200 ppm). We fed mice PLX5622-formulated chow for 21 days prior to infection and following this, for 5 or 7 days post infection. Matched control mice were fed control chow (AIN-76A) until sacrifice at dpi 5 or 7. Mice were fed either PLX5622 or AIN-76A for no longer than an additional 7 days post infection.

#### Abrogating CNS monocyte infiltration

Preventing monocyte infiltration into the virus-infected CNS was achieved using either clodronate liposomes (Liposoma, AMS) or monoclonal blocking antibodies (BioXcell, USA). Clodronate liposomes (Liposoma, AMS) were vortexed and delivered intravenously via the lateral tail vein at dpi 5 at a dose of 200 μl. Anti-Ly6C (BE0203, BioXcell, USA) and its isotype control (2A3, BioXcell, USA) were injected intraperitoneally at either (1) dpi 4, if mice were culled at dpi 5, (2) dpi 4 and 5, if mice were culled at dpi 6 or (3) dpi 5 and 6, if mice were culled at 7, at a dose of 100 or 200 μg prepared in 200 μl of sterile PBS.

#### T cell depletion

Monoclonal blocking antibodies (BioXcell, USA), anti-CD4 (BE0003-1) and anti-CD8 (BE0117) and their isotype control (LTF-2, BE0090) were injected intraperitoneally at either 1) dpi 4 and 5, if mice were culled at dpi 6 or 2) dpi 4 and 6, if mice were culled at dpi 7, in a single daily dose of 200 μg prepared in 200 μl of sterile PBS.

#### Detection of proliferating cells with BrdU

Bromodeoxyuridine (Sigma-Aldrich, USA) was injected intraperitoneally 3 h before sacrifice at a dose of 1 mg prepared in 200 µL sterile PBS.

### Quantification of viral titre using a plaque assay

Virus-susceptible Baby Hamster Kidney fibroblast (BHK) cells were used to perform a virus plaque assay as previously described [25]. BHK cells were infected with ten-fold dilutions of brain tissue homogenates for 1 h, before being replaced with an Agarose plug. Cells were incubated for a further 3 days before being fixed with 10% formalin (Sigma-Aldrich, USA) and stained with a 1% crystal violet solution (Sigma-Aldrich, USA). The plaque forming unit (PFU) per gram was calculated using the number of plaques counted, the inoculum volume, and the dilution used.

### Tissue processing and spectral cytometry

All mice were anaesthetised and transcardially perfused with ice cold sterile PBS before tissue collection. Spleens were gently mashed through a 70 μM nylon mesh sieve using a syringe plunger and the red cells in the slurry lysed with RBC lysis buffer (Invitrogen, USA). Brains were dissociated in PBS and DNase I (0.1 mg/mL, DN25, Sigma-Aldrich, USA) and collagenase type IV (1 mg/mL, C5138, Sigma-Aldrich, USA) using the gentleMACS dissociator (Miltenyi Biotec, DE). A 30%/80% Percoll gradient was subsequently used to isolate the cells from brain homogenates. After tissue processing, live cells were counted with trypan blue (0.4%) on a haemocytometer. Single cell suspensions were incubated with purified anti-CD16/32 (Biolegend, USA) and Zombie UV Fixable Viability kit (Biolegend, USA) and subsequently stained with a cocktail of fluorescently-labelled antibodies (Table 1). After surface staining, cells were washed twice and fixed in fixation buffer (Biolegend). Intracellular antibodies were used after surface staining, fixation and incubation with Cytofix/Cytoperm (BD Biosciences, USA). Anti-BrdU (3D4 or Bu20a, Biolegend, USA) was stained intranuclearly, as previously described [1]. Briefly, after cell surface staining and fixation, cells were incubated in Cytofix/Cytoperm (BD Biosciences, USA), Cytoperm Permeabilization Buffer Plus (BD Biosciences, USA) and DNase I (DN25, 30 U/sample) (Sigma-Aldrich, USA), prior to being stained with anti-BrdU.

**Table 1:** Antibodies used for flow cytometry

Antibody	Clone	Company
Surface antibodies		
anti-CD11b*	M1/70	Biolegend and BD Biosciences, USA
anti-CD11c	HL3	Biolegend
Anti-CD11c	N418	BD Biosciences, USA
anti-B220	RA3-6B2	Biolegend and BD Biosciences, USA
anti-CD8a	53-6.7	BD Biosciences, USA
anti-CX3CR1*	SA011F11	Biolegend, USA
anti-CD117	2B8	Biolegend, USA
anti-I-A/I-E	M5/114.15.2	Biolegend, USA
anti-Ly6C	HK1.4	Biolegend, USA
anti-Ly6G*	1A8	Biolegend, USA
anti-F4/80	BM8	Biolegend, USA
anti-CD4	RM4-5	Biolegend, USA
anti-Sca-1	D7	Biolegend, USA
anti-P2RY12*	S16007D	Biolegend, USA
anti-CD64*	X54-5/7.1	Biolegend, USA
anti-Siglec-H	551	Biolegend, USA
anti-TER119	TER-119	BD Biosciences, USA
anti-CD45	30-F11	BD Biosciences, USA
anti-CD3ε*	145-2C11	Biolegend and BD Biosciences, USA
anti-NK1.1*	PK136	BD Biosciences, USA
anti-CD115	AFS98	Biolegend, USA
anti-CD86	GL-1	Biolegend, USA
anti-CD48	HM48-1	Biolegend, USA
Anti-CD49d (VLA4)*	9C10 (MFR4.B)	Biolegend, USA
Anti-CD40	3/23	Biolegend, USA
Anti-Galectin-3 (Mac-2)	M3/38	Biolegend, USA
Anti-CD274 (PDL-1)	10F.9G2	Biolegend, USA
Anti-CD80	16-10A1	Biolegend, USA
Anti-H-2k^b^	AF6-88.5	Biolegend, USA
Intracellular antibodies		
Anti-CD68	FA-11	Biolegend, USA
anti-CD206	C068C2	Biolegend, USA

*Antibodies used for cell sort for scRNA analysis

Fluorescently-tagged antibodies were measured on the 5-laser Aurora, Spectral cytometer (Cytek Biosciences, USA). Spectral data was unmixed in SpectroFlo (Cytek Biosciences, USA), with unstained cells for each individual treatment and control tissue used for group-specific autofluorescence extraction. Acquired data was analysed in FlowJo (v10.8.1, BD Biosciences, USA). Quality control gating including time, single cells, non-debris/cells and Live/Dead staining was applied to exclude debris, doublets and dead cells. Cell subsets were identified using gating strategies shown in Spiteri et al., 2021 [76] and Spiteri et al., 2022 [75]. Cell numbers were quantified using cell proportions exported from FlowJo and total live cell counts. Heatmaps were applied to median fluorescent intensity (MFI) signals from populations of interest in RStudio (1.4.1717) using the R package, pheatmaps [39]. Rows and/or columns were clustered on for hierarchical clustering using the complete parameter for clustering_method() and euclidean parameter for clustering_distance().

### Tissue preparation, immunofluorescent labelling and microscopy

Tissue was isolated from mice perfused transcardially with PBS and 4% PFA. Brains were cut sagittally and placed in 4% PFA overnight, subsequent to being placed in a gradient of sucrose solutions (10%, 20%, and 30% sucrose in PBS). Tissue was frozen in optimal cutting temperature (O.C.T.) compound (Tissue-Tek, Tokyo Japan) in hexane pre-chilled in liquid nitrogen and stored at − 80 °C. Brains were cryosectioned at 8–9 μm and caught on positively-charged slides. For immunofluorescent staining, frozen sections were defrosted, air-dried, fixed in methanol, blocked with 10% fetal calf serum in 0.05% Tween 20 and TRIS-buffered saline and stained with a cocktail of primary fluorophore-conjugated antibodies for 1 h. These included anti-NS1 (clone 4G4, kindly provided by Roy Hall, The University of Queensland), anti-NeuN (Fox3, Abcam) and anti-CD11b (M1/70, Biolegend). Tissue sections were then rinsed and sealed with a coverslip using DAPI with anti-fade mounting media (Invitrogen, USA). Sections were imaged on the Olympus BX-51 microscope using a DP-70 camera and Cell Sensor software. Images were false-coloured and merged in ImageJ (1.51 s).

### 3D imaging

After overnight fixation, brains were washed in PBS. Sections (500 μm) were cut with a McIlwain tissue chopper before being incubated in methanol and then washed in PBS. Sections were stained with anti-Iba1 (SAB2500041, Sigma-Aldrich), biotinylated-anti-NS1 (4G4, Roy Hall, UQ) and anti-NeuN (Fox3, Abcam) in 2% FBS, 0.1% Triton-X and 0.01% NaN_3_ in PBS for 3 days with constant rocking. After, sections were washed in PBS and then incubated with anti-goat-CF488A (SAB4600032, Sigma-Aldrich), streptavidin-AF594 (Invitrogen) and anti-rabbit-CF647 (SAB4600352, Sigma-Aldrich) for 3 days. Sections were washed and then cleared in 20% DMSO, 40% 2,2'-thiodiethanol, 20% d-sorbitol and 0.5 M TRIS for 1 h before imaging [71]. Images were captured on the Zeiss LSM800 (Sydney Microscopy & Microanalysis, USYD) using Zen Blue software. Z-stacks were acquired with a Plan-Apochromat 20x/0.8 M27 with lasers at 405, 488, 561 and 640 nm and emission collected through 400–593 and 642–700 nm on one track and 400–551 and 577–700 nm on the other track. Laser power, gain and offset were interpolated through the z-stack to maintain signal intensity.

### RNA extraction and real-time quantitative polymerase chain reaction

Brain tissue was homogenized in TRI Reagent (Sigma Aldrich, USA) using a tissue homogenizer (TissueLyser, Qiagen, DE). The High-Capacity cDNA Reverse Transcription Kit (ThermoFisher Scientific, USA) was used to generate cDNA and the Power SYBR™ Green PCR Master Mix (ThermoFisher Scientific, USA) was used to conduct qPCR, using primers all purchased from Sigma Aldrich, USA (Table 2) on the LightCycle® 480 Instrument II (Roche, CH), as previously described [55]. Gene expression values were normalized to *Rpl13a*.

**Table 2:** Primer sequences used for qPCR

Primer	NM	Primer set sequence F 5'-3'	Primer set sequence R 5'-3'
Arg1	NM_007482	CTGACCTATGTGTCATTTGG	CATCTGGGAACTTTCCTTTC
Ccl2	NM_011333.3	CAAGATGATCCCAATGAGTAG	TTGGTGACAAAAACTACAGC
Ccl3	NM_011337	CCATATGGAGCTGACACCCC	GAGCAAAGGCTGCTGGTTTC
Ccl4	NM_013652	GGTATTCCTGACCAAAAGAG	TCCAAGTCACTCATGTACTC
Ccl5	NM_013653	TGCTCCAATCTTGCAGTCGT	GCAAGCAATGACAGGGAAGC
Ccl7	NM_013654	CTCTCTCACTCTCTTTCTCC	TCTGTAGCTCTTGAGATTCC
Cd81	NM_133655	CGTAAACAAAGACCAGATCG	GTCTCATGGAAAGTCTTCAC
Csf-1r	NM_001037859	GCCATATACAGGTACACATTC	GTGCCATTAAGAAGTACTGG
Csf-1	NM_001113529	TAGAAAGGATTCTATGCTGGG	CTCTTTGGTTGAGAGTCTAAG
Cx3cl1	NM_009142	CTTCCATTTGTGTACTCTGC	ACTCCTGGTTTAGCTGATAG
Cxcl10	NM_021274	AAAAAGGTCTAAAAGGGCTC	AATTAGGACTAGCCATCCAC
Cxcl16	NM_023158.6	CCATTCTTTATCAGGTTCCAG	CTTGAGGCAAATGTTTTTGG
Cxcl9	NM_008599	GAGGAACCCTAGTGATAAGG	GTTTGATCTCCGTTCTTCAG
Ifn-α	N/A	TGCAACCCTCCTAGACTCATT	CCAGCAGGGCGTCTTCCT
Ifn-β	N/A	ATGAGTGGTGGTTGCAGGC	TGACCTTTCAAATGCAGTAGA
Ifn-γ	NM_008337.4	GCAAAAGGATGGTGACATGA	TTCGCCTTGCTGTTGCTGA
Il-1β	NM_008361.4	TGCCACCTTTTGACAGTGATG	TGATGTGCTGCTGCGAGATT
Il-10	NM_010548.2	AAGGGTTACTTGGGTTGCCA	AAATCGATGACAGCGCCTCAG
Il-1α	NM_010554.4	CATAACCCATGATCTGGAAG	ATTCATGACAAACTTCTGCC
Il-34	NM_001135100	GTTCTTGCTGTAAACAAAGC	ATACATAGATTGCTCAGGGC
Tgf-β	NM_011577.2	GGATACCAACTATTGCTTCAG	TGTCCAGGCTCCAAATATAG
Tlr3	NM_126166.5	AATAGCATCAAAAGAAGCCG	GATGTACCTTGAATCTTCTGC
Tlr7	NM_001290758	CTTCAAGAAAGATGTCCTTGG	AAATTTGTCTCTTCCGTGTC
Tnfa	NM_013693.3	ATGGCCTCCCTCTCATCAGT	GTTTGCTACGACGTGGGCTA
Wnv	N/A	AAGTTGAGTAGACGGTGCTG	AGACGGTTCTGAGGGCTTAC

### Statistical analysis

GraphPad Prism 9.3.1 (GraphPad Software, La Jolla, CA) was used to apply non-parametric statistical tests to data. Comparison of two groups was conducted using Mann–Whitney test, and three or more groups were compared using a Kruskal–Wallis test with a Dunn’s multiple comparison test. When two independent variables and three or more groups were being compared a Two-way ANOVA and a Šídák's or Tukey’s multiple comparisons test was used. Error bars are shown as standard error of the mean (SEM).

## Results

### Single-cell transcriptomic analysis on sorted myeloid cells from the virus-infected CNS

**Fig. 1:**
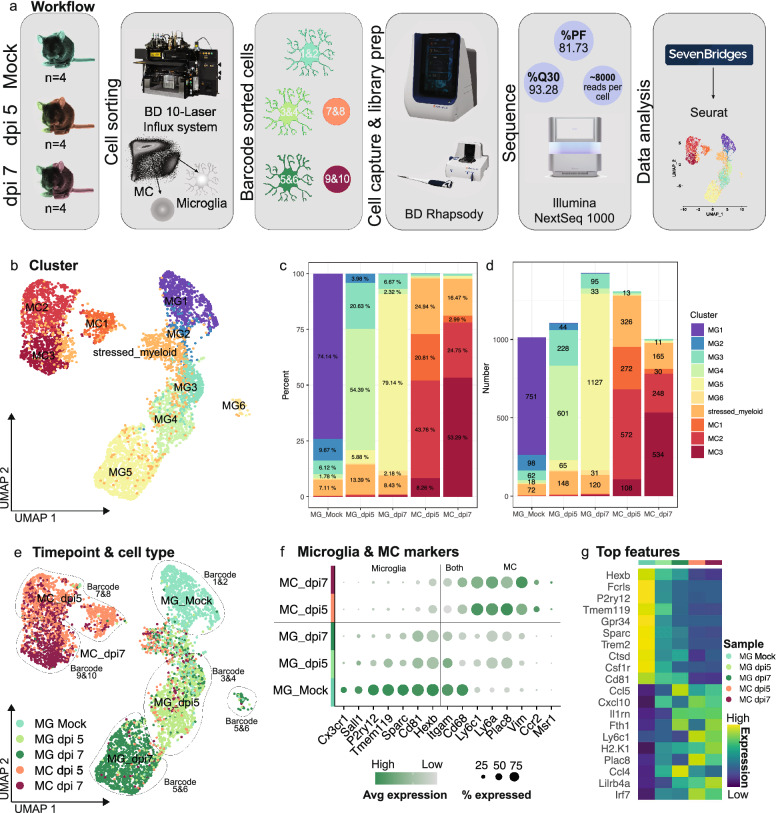
Single-cell RNA sequencing analysis on resident and infiltrating myeloid cells from the virus-infected CNS. **a** Schematic showing scRNA-seq pipeline. Microglia from mock-infected and microglia and MCs from WNV-infected brains at dpi 5 and 7 were flow cytometrically sorted, barcoded and pooled in equal numbers for cell capture on the BD Rhapsody. Libraries were sequenced on the Illumina NextSeq 1000 and processed in SevenBridges and Seurat. **b** UMAP plot showing 6 microglia and 3 MC clusters. **c, d** Percent (**c**) and number (**d**) of each microglia and MC cluster out of total microglia and MC populations in mock-infected and infected brains at dpi 5 and 7. **e** UMAP plot coloured by cell type and timepoint and overlaid with sample identifiers/barcode numbers. **f** Dot plot showing the expression of nominal microglia and MC genes in total microglia and MC populations in mock-infected and infected brains at dpi 5 and 7. **g** Heatmap showing the top differentially expressed genes in total microglia and MC populations in mock-infected and infected brains at dpi 5 and 7. Data is presented from one independent experiment with four mice per group

To define microglial responses during severe neuroinflammation we performed scRNA-seq on sorted cells from brains in lethal progression of WNE (Fig. 1). Live microglia from mock-infected, and microglia and MCs from WNV-infected brains at 5- and 7-days post infection (dpi) (early and late infection) were flow cytometrically sorted (See Additional file 1 for gating strategy for cell sorting), barcoded and pooled in equal numbers for cell capture on the BD Rhapsody (Fig. 1a). As microglia do not downregulate cell-surface expression of the nominal homeostatic marker, P2RY12, following WNV infection [76], they were identified as Ly6G^−^, CD49d^lo^, P2RY12^+^, CD11b^+^ and CX3CR1^+^ (Additional file 1). MCs were gated as Ly6G^−^, CD49d^hi^, P2RY12^lo^, NK1.1^−^, CD3e^−^, CD11b^+^ CD64^+^ and CX3CR1^+^, identifying both Ly6C^hi^ and Ly6C^lo^ macrophages [76]. Circulating monocytes were not identified, however the presence of these cells is expected to be low in the brain due to transcardiac perfusion prior to tissue collection. Each barcode contained a single cell type (*i.e.,* microglia or MC) from a single timepoint (i.e., mock-infected, dpi 5 or dpi 7), enabling precise identification of the origin of each sequenced cell. The BD Immune response panel, consisting of 397 genes and a panel of 67 custom genes, including 6 mitochondrial genes, were sequenced on the Illumina NextSeq 1000 (See Additional file 2 for full gene list). Sequencing reads were processed in SevenBridges and PCA, and UMAP dimensionality reduction was performed on processed data using Seurat [8, 33, 69, 81] (Fig. 1a).

Dimensionality reduction on 5851 cells identified 6 microglia clusters (MG1-6), 3 MC (MC1-3) clusters and a ‘stressed’ myeloid population, across 3 timepoints (Fig. 1b). This myeloid population was designated ‘stressed’ as these cells primarily expressed mitochondrial genes and were present in all barcoded samples, suggesting that this cluster did not represent a particular cell type but rather a shared transcriptomic profile adopted by microglia and MCs that were stressed or undergoing apoptosis during tissue digestion or scRNA-seq cell capture [57] (Fig. 1b–d).

Notably, barcodes used to stain sorted microglia (barcode 1–6) and MCs (barcode 7–10) prior to cell capture contained little to no contamination of the contrasting cell types (Fig. 1c–e), as defined computationally by nominal microglia and MCs markers (Fig. 1f), indicating accurate identification and gating of populations during cell sorting. Further confirming the identity of our sorted populations, MCs expressed nominal MC markers including *Ly6c1, Ly6a, Plac8* and *Vim*, while all microglia clusters expressed *Hexb* and the recently defined microglial-enriched and inflammatory stable marker *Cd81* [107] (Fig. 1f, g). Strikingly, microglia at dpi 5 and 7 substantially downregulated *microglia-specific* genes *Sall1, P2ry12, Tmem119* and *Sparc* and upregulated MC marker genes, *Ly6a* and *Plac8* (Fig. 1f, g)*.* This highlights the importance of strategic barcoding approaches, as shown here, as microglia can adopt substantially disparate transcriptional profiles from homeostatic origins, that would confound determination of cell type identification. It also highlights the disparity between genes and cell-surface protein expression of P2RY12/*P2ry12*.

Intriguingly, different microglia and MC clusters were predominant at each timepoint. Thus, MG1 was the prominent cluster in mock-infected brains, while MG3, MG4 and MC2 were the main clusters in dpi 5 brains and MG5 and MC3 clusters were most prominent in dpi 7 brains (Fig. 1b–e). MG2, MG3 and MC2, however, were prominent across two timepoints, i.e., MG2 was present in mock-infected and to a lesser extent in dpi 5 brains, MG3 was present in mock-infected, but increased in dpi 5 brains, while MC2 was present in dpi 5 and to a lesser extent in dpi 7 brains (Fig. 1b–e), potentially representing a phenotype transition from one timepoint to another. Overall however, this indicates that myeloid populations adopt unique and global transcriptomic profiles at particular timepoints in the progression of WNE. Extensive cytometric profiling of these cells has also demonstrated global proteomic changes with disease progression [76], mirroring these timepoint-specific transcriptomic changes.

### 3.1 Microglia and MCs adopt timepoint-specific populations connected along a trajectory

**Fig. 2:**
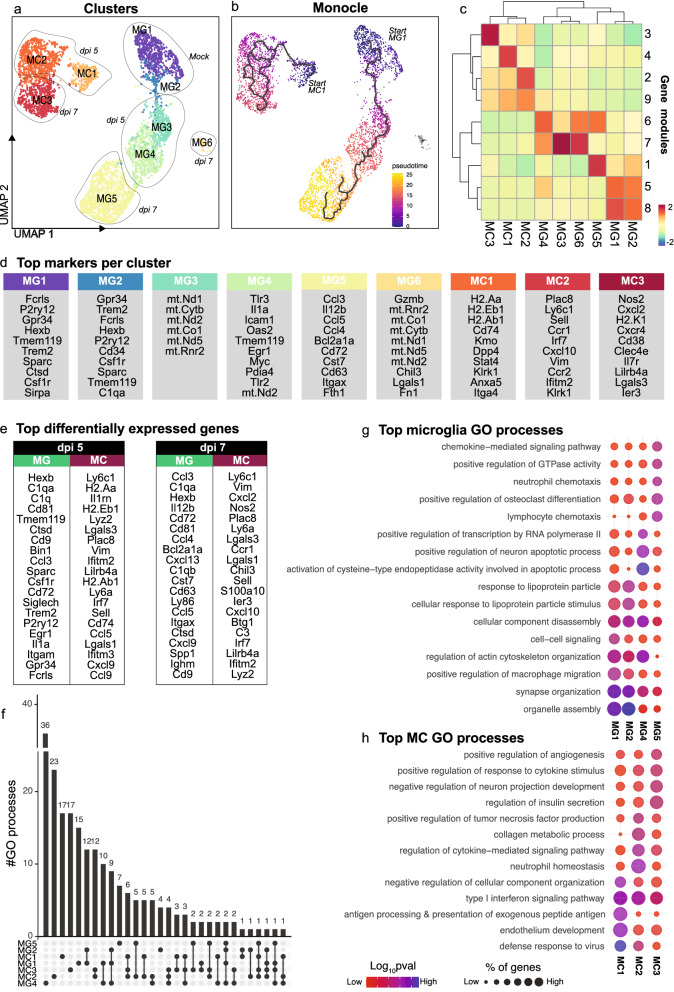
Microglia and MCs adopt unique timepoint-specific transcriptomes connected along a trajectory. **a** UMAP plot showing 6 microglia and 3 MC clusters. **b** UMAP plot coloured by pseudotime, determined using trajectory analysis with Monocle3. Increased pseudotime indicates further distance from starting cells (i.e., MG1 and MC1). **c** Clustered heatmap showing expression of gene modules in each microglia and MC cluster. **d** Top differentially-expressed genes per cluster. **e** Top differentially expressed microglia and MC genes at dpi 5 and 7. **f** UpSet plot showing overlap in the number of gene ontology (GO) biological processes enriched in microglia and MCs clusters. GO processes common to two or more clusters are denoted by a connecting line. **g, h** Dot plot showing the top 5 GO processes associated with each microglia (**g**) and MC (**h**) cluster. Data is presented from one independent experiment with four mice per group

To investigate the relationship between the different timepoint-specific transcriptomic profiles adopted by the resident and infiltrating myeloid populations in the CNS, we firstly performed a trajectory-based analysis using Monocle3 (Fig. 2a, b). Monocle3 identifies genes that change as a function of *pseudotime* and orders single cells along a learned trajectory, informing the total amount of transcriptional change a cell undergoes from the starting state and the relatedness or distance between these cell states [10, 58, 86]. For this analysis we removed the stressed myeloid population from each timepoint, since this did not represent a unique cell type.

To run Monocle3 we set the beginning of the trajectory to two cell states (or “roots”), representing the *start* of the two independent myeloid lineages (i.e., microglia and MCs). We used MG1 *i.e.,* microglia from mock-infected brains and MC1 *i.e.,* the MC cluster found in dpi 5 brains, but with very low representation in dpi 7 brains, which likely represents the earliest infiltrating MC population analysed. Interestingly, microglia and MC clusters were assigned to two separate branched trajectories with clusters placed broadly speaking in the precise order of disease progression, i.e., from mock-infected to dpi 7 cells: (1) MG1, MG2, MG3, MG4, and MG5 and (2) MC1, MC2 and MC3 (Fig. 2a, b). Notably, MG6 was not connected with the microglia or MC trajectory, suggesting that this is a distinct phenotype, despite expressing microglia markers *Hexb* and *Cd81* (Additional file 3). Overall, however, this analysis demonstrates that during the course of infection, both microglia and MCs progressively differentiate into unique transcriptional states over time. This is particularly evident in microglia from the correspondence of the pseudotime trajectory with the appearance of these populations in real time (Fig. 2a, b). The genes informing these transcriptional changes, i.e., those that are gained or lost with disease progression and pseudotime by each cluster (Fig. 2c) was determined by generating co-regulated modules of differentially-expressed genes in Monocle3 (Fig. 2c and Additional file 2). Importantly, each cluster was defined by a unique set of these gene modules, confirming the cell-specific transcriptional alterations and associated functions adopted with progression of CNS infection. Notwithstanding, microglia and MC clusters did not share a single gene module, highlighting their unique transcriptional identities in WNE (Fig. 2c). Supporting this, setting the starting cell state to just MG1 or just MC1 demonstrated no connection between the opposite cell type (Additional file 3).

### Defining microglial and MC responses to CNS viral infection with scRNA-seq

To understand the function of these timepoint-specific microglia and MC clusters we inspected populations for their top differentially-expressed markers (relative to all other clusters) and analysed these for associated gene ontology (GO) enrichment processes (Fig. 2d–h).

Of the microglial clusters, MG1 and MG2 were most prominent in the mock-infected brain, and were enriched for nominal homeostatic microglia genes, *Fcrls, P2ry12, Gpr34, Hexb, Tmem119, Trem2, Sparc, Ctsd and Csf1r* (Fig. 2d and Additional file 4 for a list of all top markers). These populations shared the highest number of intersecting GO processes (a total of 12) (Fig. 2f), and were associated with the regulation and organization of organelles, synapses, actin cytoskeleton and cellular components, as well as response to lipoproteins, amyloid-beta clearance, phagocytosis and the migration, development and differentiation of glial cells for neuronal signaling, myelination and homeostasis (Fig. 2g and Additional file 5 for a list of all processes associated with each cluster).

In the WNV-infected brain, microglia adopted two unique functional phenotypes, the MG4 phenotype at dpi 5 and the MG5 at dpi 7. Since MG3 and MG6 expressed mainly mitochondrial genes at both timepoints (Fig. 2a, d), these may represent 2 additional, presumptively ‘stressed’ phenotypes. Alternatively these populations may be dying microglia or microglia that adopt unique functions at specific timepoints, since neither microglia from mock-infected animals, nor MCs from infected animals exhibited this phenotype. Functional enrichment analysis, however, was not performed on these populations, as their top markers consisted of 10 or less genes, which were primarily mitochondrial. Nonetheless, differentially-expressed microglia markers suggest that microglia progress from a homeostatic (MG1 and MG2) to an *anti-viral* population at dpi 5, with MG4 enriched for *Tlr3, Oas2, Tlr7, Nlrp3, Nod1* and *Nfkb1,* and then into an *immune cell-recruiting* population at dpi 7, with MG5 enriched for *Ccl3, Ccl4, Ccl5, Cxcl13 and Cxcl16* (Fig. 2d and Additional files 4 and 6). Consistent with this, MG4 (*anti-viral* microglia) was associated with the regulation of viral processes, in addition to increased transcription from an RNA polymerase II promoter, leukocyte migration and the positive regulation of apoptosis (including of neurons), as well as IL-1β, IL-6, IL-8, tumour necrosis factor (TNF), IFN-β and nitric oxide (NO) production (Fig. 2g and Additional file 5). MG4 (*anti-viral* microglia) was also associated with CNS regulatory functions that were enriched in MG1 and MG2 (predominantly mock-infected microglia), as shown by the high number of intersecting biological processes connecting MG1 MG2, and MG4 on the Upset plot (Fig. 2f). These processes include synapse, organelle and actin filament organization, cellular component disassembly and gliogenesis, indicating a transition from homeostatic to an anti-viral response at dpi 5 in MG4 (*anti-viral* microglia) (Fig. 2g). Interestingly, by dpi 7, microglia were associated with the least number of unique GO processes (Fig. 2f), corresponding with the lowest number of top differentially expressed genes out of the non-stressed microglia clusters (Additional file 4). Nonetheless, these top genes implicated MG5 (*immune-cell recruiting* microglia at dpi 7) in lymphocyte and neutrophil chemotaxis and responses to TNF, IL-1 and IFN-γ (Fig. 2g and Additional file 5). Overall, this work suggests that microglia adopt an early anti-viral response by dpi 5 and with further disease progression these cells produce increasing chemotactic signals for immune cell recruitment.

While MC and microglial populations do not cluster together via UMAP (Fig. 2a), are unrelated via trajectory analysis (Fig. 2b) and show unique differentially expressed markers (Fig. 2c–e), GO analysis nevertheless demonstrated functional overlap between these cell types. All MC clusters expressed anti-viral interferon-stimulated genes (ISGs) including *Ifitm2* and *Ifitm3*, with MC1 and MC3 also expressing *Oas2* (Fig. 2d and Additional files 4 and 6), suggesting shared anti-viral activity by MC populations. Other top MC markers suggest that MCs change from an *antigen-presenting* (MC1) to an *immune cell-recruiting* (MC2 and MC3) and *inflammatory* phenotype (MC3) at dpi 5 and 7, respectively (Fig. 2d and Additional file 4). Indeed, functional enrichment analysis of these top differentially-expressed markers, show MC1 is involved in antigen processing and presentation of peptide antigen via MHC-II, endothelial development, positive and negative regulation of T cell activation and neutrophil migration (Fig. 2h and Additional file 5). On the other hand, MC2 found in both dpi 5 and dpi 7 brains, was involved in neutrophil homeostasis, TNF, IL17 and IL-1β production, apoptotic cell clearance, monocyte chemotaxis, NK cell differentiation, T cell migration and proliferation, response to TNF, positive regulation of apoptosis and wound healing, and programmed necrotic cell death (Fig. 2h and Additional file 5). In contrast, MC3, predominantly found in dpi 7 brains, was associated with T cell apoptosis, monocyte chemotaxis, negative regulation of cell death, apoptotic cell clearance, vascular endothelial growth factor production and negative regulation of neuron development (Fig. 2h and Additional file 5). Compared to predominant microglia clusters in the WNV-infected brain (i.e., MG3-6), MC clusters 1–3 had more overlapping functions, with all MC populations involved in the regulation of viral life cycle, type I interferon signaling pathway and responses to IFN-γ (Fig. 2h and Additional file 5). This is demonstrated by the higher number of connected biological processes between MC clusters on the Upset plot (Fig. 2f). Overall, MCs appear to be primarily involved in antigen presentation (MC1) in the early phase of infection, with these cells adopting an immune-cell recruiting (MC2 and MC3) and inflammatory response (MC3) by dpi 7.

### Microglia transition from an early *anti-viral* to an* immune-cell recruiting* phenotype in lethal viral infection

**Fig. 3:**
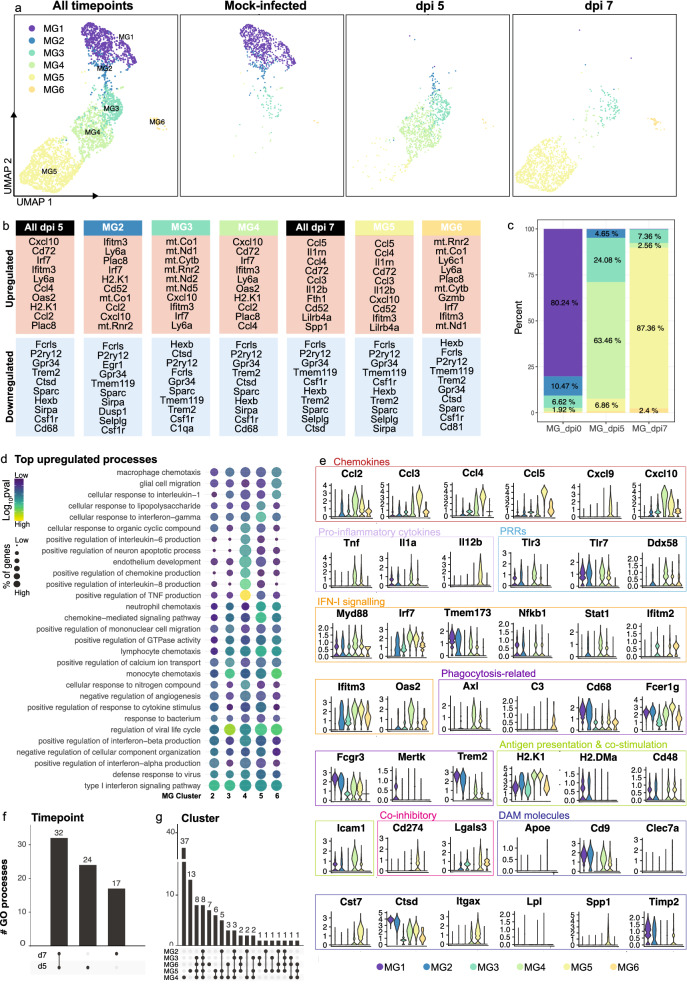
Microglial transcriptomic response kinetics in lethal viral infection. **a** UMAP plots showing MG1-6 in mock- and WNV-infected brains. **b** Top 10 up- and down-regulated genes in total microglia or in MG2-6 at dpi 5 and 7, relative to MG1 in mock-infected brains. **c** Frequency of microglia clusters out of total microglia in mock-infected and infected brains at dpi 5 and 7. **d** Dot plot showing the top 5 gene ontology (GO) processes associated with upregulated genes in MG2-6, relative to MG1. **e** Violin plots showing the expression of selective genes in MG1-6. **f, g** UpSet plots showing overlap in the number of GO biological processes upregulated by total microglia populations at dpi 5 and 7 (**f**) and microglia clusters, MG2-6 (**g**). GO processes common to two or more samples/clusters are denoted by a connecting line. Data is presented from one independent experiment with four mice per group

Next, we analysed timepoint-specific microglial clusters for their up- and downregulated genes, to investigate microglial transcriptomic response kinetics with disease progression (Fig. 3). For this analysis, changes in gene expression in microglial clusters MG2-6 were compared to MG1, the prominent population in the mock-infected brain (Fig. 3a).

Relative to MG1, microglial clusters, MG3-6, found in WNV-infected brains, downregulated 67 common genes (Fig. 3b and Additional file 7 for a list of up- and down-regulated genes). Functional enrichment analysis of downregulated genes demonstrates the loss of homeostatic processes during infection, with genes downregulated by MG3-6 commonly associated with the organization of actin filaments, actin cytoskeleton, synapses and the endomembrane system, astrocyte differentiation and responses to lipoprotein particles (see Additional file 8 for processes associated with downregulated genes). These processes are enriched in homeostatic populations, MG1 and MG2, but clearly lost by dpi 5 and 7.

Interestingly, of the upregulated genes, all microglia clusters were associated with the regulation of virus replication and immune cell recruitment. All microglia clusters upregulated *Ccl2, Cxcl10, Irf7, Oas2, Ifitm3* (Fig. 3b and Additional file 7) and were associated with the response to virus, bacterium and IFN-γ, the regulation of viral life cycle and calcium ion transport, monocyte chemotaxis and type 1 IFN signaling pathway (Fig. 3d and Additional file 9 for processes associated with upregulated genes). Thus, while stressed microglia populations, MG3 and MG6 differentially expressed mitochondrial genes relative to all the other myeloid clusters (Fig. 2d), they also upregulated immune response genes (Fig. 3b), supporting a functionally important role for these populations and suggesting that increased mitochondrial expression in this context may potentially indicate cells with a higher energy demand, rather than a stressed or dying population [20].

While all microglial clusters demonstrate anti-viral and immune-cell recruiting responses, particular clusters demonstrate a more prominent role in each of these. For instance, the expression profile of MG2, a population found in the mock-infected and dpi 5 brain, suggests this may represent an early anti-viral phenotype, coinciding with neuronal WNV infection from dpi 5 [25, 76]. MG2 (early *anti-viral* microglia) was associated with type 1 interferon signaling and production and regulation of viral life cycle, due to their upregulation of relevant genes including *Tlr3, Tlr7, Ddx58* (RIG-1*), Myd88, Irf7, Tmem173* (STING*), Stat1* and *Ifitm3* (Fig. 3b, d, e and Additional file 7 and 9)*.* This anti-viral response, however, is heightened in MG4 (*anti-viral* microglia), the predominant microglia population at dpi 5, which more highly expressed the viral RNA sensors, *Tlr3, Tlr7* and *Ddx58* (*Rig-1*), as well as anti-viral ISG, *Oas2* (Fig. 3e). This population, along with MG5 (*immune-cell recruiting* microglia), had the highest number of upregulated genes and associated biological processes (Fig. 3g and Additional files 7 and 9). In addition to anti-viral responses, MG4 was also associated with pro-inflammatory functions, including positive regulation of TNF, IL-1α, IL-6 and IL-8 (Fig. 3d and Additional file 9). Thus, while microglia may be protective due to their anti-viral status they can also simultaneously promote neuroinflammation.

**Fig. 4:**
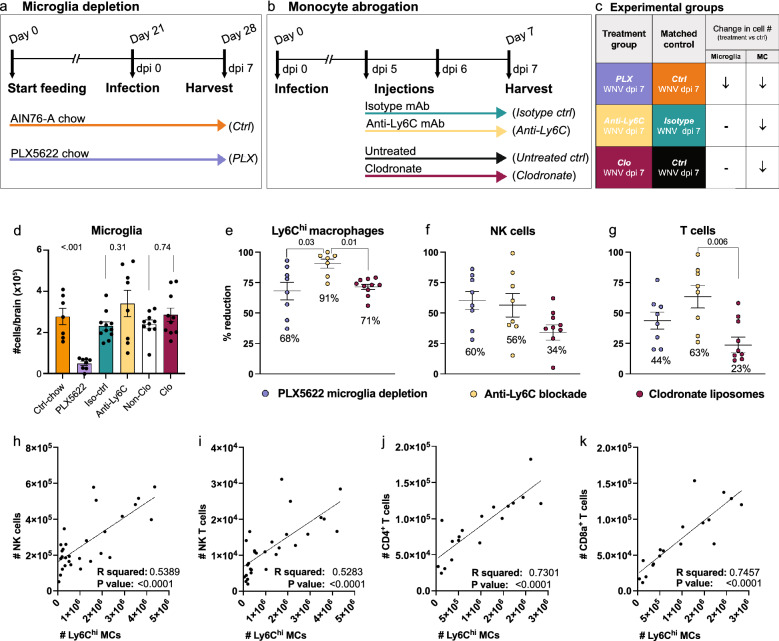
Microglia and MC depletion abrogates the infiltration of NK and T cells into the virus-infected CNS. **a, b** Schematic showing experimental regimens used to deplete microglia (**a**) or MCs (**b**) in the CNS. Three principal groups were set up, mice were (1) fed PLX5622-formulated chow 21 days prior to infection and until sacrifice to deplete microglia (**a**), (2) injected with an anti-Ly6C monoclonal antibody (mAb) at dpi 5 and 6 or (3) injected with clodronate liposomes at dpi 5 to reduce MC CNS infiltration and sacrificed at dpi 7 (**b**). **c** Summary table showing the three experimental groups set up to reduce microglia and/or MCs in the infected CNS and the three relevant controls. **d-g** Number of microglia (**d**) and percent of Ly6C^hi^ macrophages (**e**), NK cells (**f**) and T cells (**g**) reduced in mice treated with PLX5622, anti-Ly6C and clodronate liposomes, relative to control chow-fed (AIN-76A), isotype-treated and untreated mice, respectively. **h–k** Correlation analysis between the number of Ly6C^hi^ macrophages and the number of NK cells (**h**), NK T cells (**i**), CD4^+^ T cells (**j**) and CD8a^+^ T cells (**k**) in WNV-infected mice at dpi 7. Data is presented as mean ± SEM from at least two independent experiments with at least seven mice per group

Over the disease course, MG4 (*anti-viral* microglia) altered their transcriptomic phenotype to become MG5 (*immune-cell recruiting* microglia) by dpi 7 (Figs. 2a, b, 4a). While dpi 7 microglia adopt a unique profile, compared to dpi 5 microglia, these populations have more shared than unique biological processes (Fig. 3f). MG5 microglia increased their expression of chemotactic signals, *Ccl3, Ccl4, Ccl5* and *Cxcl9* for the enhanced recruitment of peripheral immune cells relative to the other microglia clusters (Fig. 3d, e and Additional files 6 and 9). MG5 also uniquely expressed *Il-12b* (Fig. 3e), likely to promote the differentiation of naïve CD4^+^ T cells to T helper 1 cells [87] and/or in responses to T cell-derived IFN-γ in the CNS [45]. Notably, MG4 and MG5 also upregulated *H2-k1* (Fig. 3b, e and Additional file 7), another IFN-γ-inducible gene [89, 109], potentially to enhance cross-presentation to CD8^+^ T cells. Thus, early microglia clusters demonstrate a prominent anti-viral role, while later microglia clusters appear to have an enhanced role in leukocyte migration to the brain. Indeed, using an alternate trajectory analysis tool called Slingshot [80] just on microglial clusters demonstrated two “lineages”, (1) MG1, MG2, MG3, MG4 and MG5 and (2) MG1, MG2, MG3 and MG6, suggesting that upon infection microglia may adopt (1) functionally adaptive, *i.e.,* anti-viral response (MG4) and then immune cell recruiting (MG5) or (2) stressed/dying response, differentially expressing mitochondrial genes (MG3 and MG6) with time (Additional file 3).

### Microglia and Ly6C^hi^ MCs promote immune cell recruitment into the virus-infected CNS

Single-cell RNA sequencing identified a role for microglia and MCs in peripheral immune cell recruitment, viral clearance, antigen presentation and T cell activation following CNS infection. To investigate these processes more mechanistically, we differentially depleted these cells in the WNV-infected CNS and examined single-cell brain suspensions by high-dimensional spectral cytometry for changes in immune cell infiltrates, viral loads and T cell responses. To deplete microglia by 85–90%, mice were treated with PLX5622 in a standard rodent chow (AIN76-A) for 21 days prior to infection and subsequently until sacrifice at dpi 7 (Fig. 4a, c, d) [75]. To abrogate Ly6C^hi^ inflammatory monocyte infiltration without affecting microglial numbers, we injected mice with either a monoclonal antibody (mAb) targeting Ly6C at dpi 5 and 6 or clodronate-encapsulated liposomes at dpi 5 (Fig. 4b–e) [75, 76, 83]. This reduced the numbers of MCs at dpi 7 by 91% and 71%, respectively (Fig. 4e).

Microglia depletion with PLX5622 results in a substantial reduction in the recruitment of peripheral NK and T cells into the CNS, supporting a role for microglia in immune cell recruitment [75]. However, PLX5622 also reduces peripheral monocyte production and CNS MC infiltration by 73% at dpi 5 [75] and 68% at dpi 7 (Fig. 4e). Therefore, to determine the contribution of MCs to the recruitment of peripheral NK and T cells independently of microglia, we examined the CNS of anti-Ly6C- and clodronate-treated animals.

Intriguingly, blocking MC infiltration into the CNS in these animals also reduced the NK and T cell recruitment (Fig. 4f, g), with increasing numbers of Ly6C^hi^ MCs directly correlating with peripheral immune cell numbers in the CNS (Fig. 4h–k), suggesting that MCs promote CNS infiltration. The greater reduction in NK and T cell recruitment in the brain of anti-Ly6C-treated mice, relative to clodronate-treated mice may be explained by the 20% further decrease in MCs in the anti-Ly6C group (Fig. 4e–g). However, it should be noted that anti-Ly6C likely targets Ly6C-expressing T and NK cells, thereby also directly inhibiting their CNS infiltration. Nonetheless, treatment with clodronate liposomes, which targets circulating monocytes and phagocytic cells in the BM more specifically [93–95], resulted in a 23–34% reduction in NK and T cells in the CNS (Fig. 4f, g), strongly supporting the contribution of MCs to immune cell recruitment. This is consistent with the high differential expression of NK and T cell chemoattractants *Ccl5, Cxcl9, Cxcl10* and *Cxcl16* by MC clusters 1–3 (Fig. 2d, e and Additional file 4). Notably, reducing both microglia and MCs in the WNV-infected CNS with PLX5622 reduced the immune cell infiltrate by two-fold more than MC depletion alone with clodronate (Fig. 4d–g), suggesting that microglia and MCs share an overlapping role in NK and T cell trafficking. This is supported by the upregulation of *Cxcl10* by all disease-related microglia clusters and the high expression of NK and T cell chemoattractants, *Ccl3, Ccl4, Ccl5, Cxcl9 and Cxcl16* at dpi 7 by MG5 (Fig. 3b, e and Additional files 4, 6 and 7). Importantly, using qPCR to confirm scRNA-seq data, many of these genes are also significantly reduced upon depletion of microglia and/or MCs in the whole brain with PLX5622 or anti-Ly6C treatment at dpi 5 and 7 (Additional file 10). Genes expressed by microglia or MCs in scRNA-seq data and subsequently not reduced in whole brains with their depletion may be a result of the increased expression by alternative cell subsets to compensate for microglia and/or MC depletion (Additional file 10). This highlights the need for additional tools to study these cells beyond cell depletion. Nonetheless, these data suggest that microglia and MCs have an overlapping role in the expression of immune-cell recruiting chemokines for cellular recruitment into the infected CNS.

### Microglia and monocyte depletion impairs T cell responses in the infected brain

**Fig. 5:**
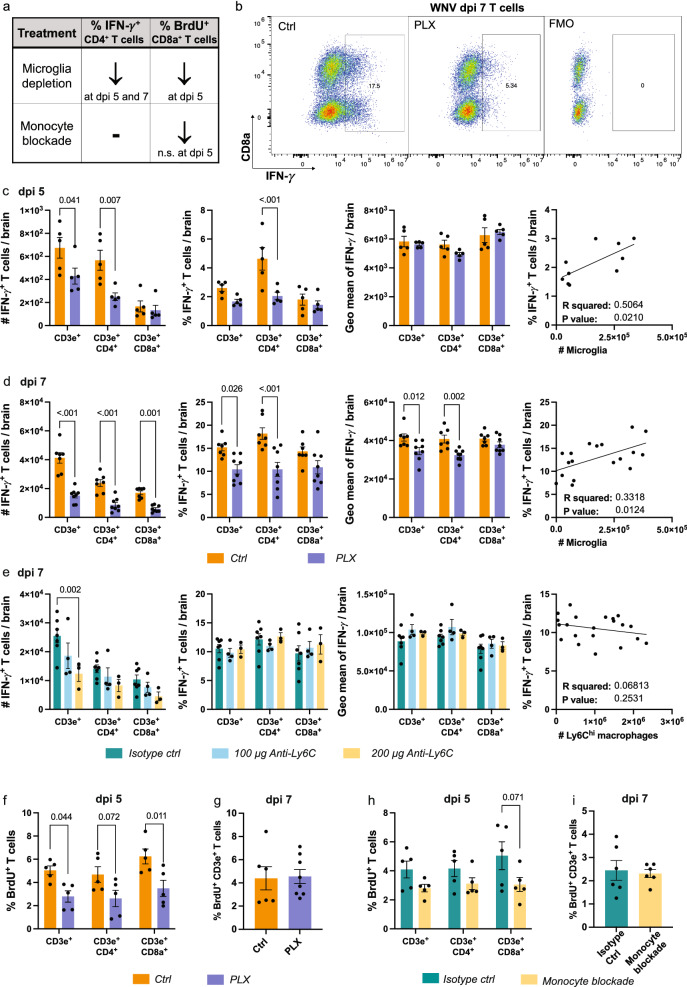
Microglia and MCs differentially promote CD4^+^ T cell IFN-γ production and CD8^+^ T cell proliferation. **a** Summary table showing changes in the frequency of IFN-γ^+^ and BrdU^+^ T cells with microglia and MC depletion in WNV-infected mice. **b** Flow cytometric dot plots showing the expression of IFN-γ in fully-stained samples and a fluorescence-minus-one (FMO, *i.e.*, without anti-IFN-γ) in non-stimulated T cells from mice fed control (*Ctrl*) or PLX5622-formulated chow (*PLX*) until sacrifice at dpi 7. **c**, **d** Number and frequency IFN-γ-producing T cells, geometric mean of IFN-γ in T cells and correlation analysis between the number of microglia and frequency of IFN-γ-producing T cells from single-cell brain suspensions from *Ctrl* and *PLX* mice sacrificed at dpi 5 (**c**) or dpi 7 (**d**)**. e** Number and frequency IFN-γ-producing T cells, geometric mean of IFN-γ in T cells and correlation analysis between the number of Ly6C^hi^ MCs and frequency of IFN-γ-producing T cells from single-cell brain suspensions from anti-Ly6C-treated mice sacrificed at dpi 7 (**e**). **f–i** Frequency of BrdU^+^ proliferating T cells in WNV-infected mice treated with PLX5622 and culled at dpi 5 (**f**) or 7 (**g**) or in WNV-infected mice treated with monoclonal antibodies to block monocyte infiltration and culled at dpi 5 (**h**) or 7 (**i**)**.** Data is presented as mean ± SEM from one or two independent experiments with 3–8 mice per group

As well as T cell migration, microglia and MC scRNA-seq clusters were enriched for T cell co-stimulation markers and processes associated with T cell proliferation, differentiation and activation (Additional file 5). Therefore, to investigate the differential contribution of microglia and MCs to T cell responses in the CNS, we stained single-cell brain suspensions for IFN-γ and BrdU to measure T cell effector functions and proliferation in microglia- and/or MC-depleted brains (Fig. 5).

Intriguingly, PLX5622 treatment resulted in the reduced production of IFN-γ by CD4^+^ T cells at dpi 5 and 7 (Fig. 5a, c, d). The frequency of T cells expressing IFN-γ also corresponded with microglia number in the CNS (Fig. 5c, d), suggesting an association of microglia with T cell responses.

To determine whether microglia and/or MCs were responsible for the reduction in IFN-γ-producing CD4^+^ T cells with PLX5622, we treated mice with two doses of anti-Ly6C, *i.e.*, 100 μg and 200 μg at dpi 5 and 6 (Fig. 5e), to abrogate MC infiltration by 50% and 91%, respectively, without affecting microglial numbers. In these experiments we used anti-Ly6C instead of clodronate, as anti-Ly6C reduced NK and T cell recruitment to frequencies that were more similar to *PLX* mice. Like PLX5622 treatment, anti-Ly6C reduced the number of IFN-γ^+^ T cells in the CNS, corresponding with the reduction in total T cell infiltration (Figs. 4g and 5e). While T cell infiltration was further reduced with a higher dose of anti-Ly6C, this had no effect on the production of IFN-γ by remaining T cells (Fig. 5e). Taken together, these data suggest that microglia and not MCs are responsible for promoting CD4^+^ T cell effector functions in the early and later phase of disease in PLX5622-treated mice, despite scRNA-seq MC clusters being more highly enriched for T cell responses. This could, however, suggest that microglia are required for MC maturation in the CNS for effective T cell responses. This would be consistent with the high differential expression of MHC-II genes by MCs, relative to microglia, with *H2-Aa, H2-Ed1, H2-Ab1* and *Cd74* highly differentially expressed by MC1 in untreated mice (Fig. 2d).

Next, we investigated whether differential myeloid cell depletion affected T cell proliferation by injecting BrdU 3 h prior to tissue collection (Fig. 5f–i). Interestingly, microglia and MC depletion with PLX5622 in the CNS reduced the proliferative capacity of both T cell subsets at dpi 5 by 44%, although this was significant only for CD8a^+^ T cells (Fig. 5f). At dpi 7, however, PLX5622 had no effect on this (Fig. 5g). Similarly, anti-Ly6C-mediated MC depletion non-significantly reduced CD4^+^ and CD8a^+^ T cell proliferation at dpi 5 by 25% and 39%, respectively and had no effect at dpi 7 (Fig. 5h, i). Together, this data suggests that MCs are required early in infection for CD8^+^ T cell proliferation. This is because the significant reduction in CD8^+^ T cell proliferation at dpi 5 in PLX5622-treated mice was recapitulated in anti-Ly6C-treated mice by a similar amount (~ 40%). Alternatively, both microglia and MCs may be required for T cell proliferation, however, the development of more specific microglia depletion methods are needed to elucidate this. Notably however, splenic T cell proliferation, which increases during WNV-infection, was not reduced with microglia and MC depletion with PLX5622, suggesting that these cells have no effect on peripheral T cell priming by CNS-derived antigen (Additional file 11).

### Microglia and MCs differentially express T cell co-stimulatory and co-inhibitory markers

**Fig. 6:**
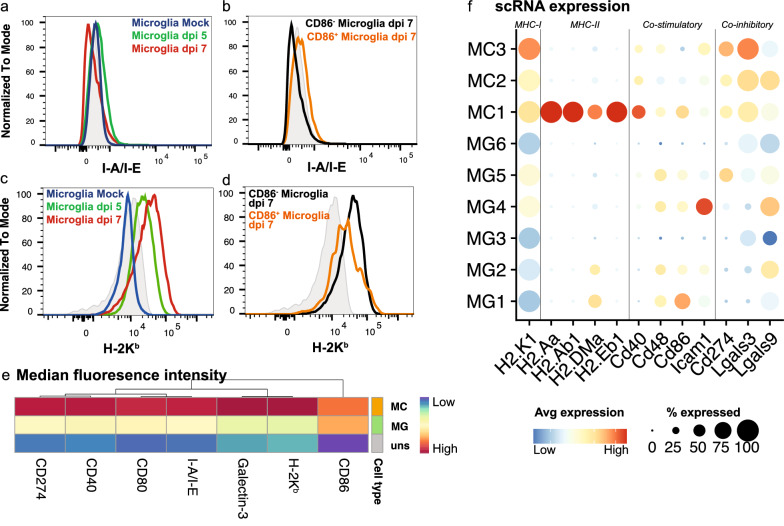
MCs express T cell co-stimulatory and co-inhibitory markers more highly than microglia in lethal CNS infection. **a-d** Histograms showing the expression of I-A/I-E (MHC-II) (**a, b**) and H-2 K^b^ (MHC-I) (**c, d**) on total microglia in mock-infected and infected brains at dpi 5 and 7 (**a, c**) and on CD86^+^ and CD86^−^ microglia at dpi 7 (**b, d**). **e** Heatmap showing the geometric mean of MHC molecules and T cell co-stimulatory and co-inhibitory markers on total MC and microglia populations at dpi 7. Hierarchical clustering was performed on both rows and columns. **f** Dot plot showing the expression of MHC and T cell co-stimulatory and co-inhibitory genes in microglia and MC clusters from scRNA-seq data

To further investigate T cell activation capabilities by microglia and MCs, we stained single-cell brain suspensions for cytometric analysis of MHC molecules and T cell co-stimulatory (antigen presenting molecules (APC)) and co-inhibitory markers (Fig. 6). Intriguingly, while microglia promote IFN-γ production by CD4^+^ T cells (Fig. 5c, d), they do not express I-A/1-E (MHC-II) or *H2-Aa, H2-Ab1, H2-DMa* and *H2-Eb1* (MHC-II molecules) during infection (Fig. 6a, f). Instead, microglia may activate these cells by producing particular cytokines. Indeed, we have demonstrated the exclusive expression of *Il-12b* by MG5 at a scRNA-seq level (Fig. 3e), as well as at a protein level with cytometric analysis [76]. This molecule is associated with promoting the differentiation of naive CD4^+^ T cells into T helper 1 cells. Alternatively, microglia may also enhance antigen presentation to CD4^+^ T cell by promoting MC maturation in the CNS, since MCs more highly express MHC-II genes, as well as co-stimulatory markers CD80, CD86/*Cd86* and CD40/*Cd40*, compared to microglia (Fig. 6e, f). However, and notably, while MCs had a higher expression of co-stimulatory markers, they have a higher expression of co-inhibitory molecules, CD274/*Cd274 (Pd-l1)*, Galectin-3/*Lgals3* and *Lgals9* (Fig. 6e, f).

In contrast to MHC-II molecules, microglia upregulated H-2 K^b^(MHC-I) and *H2-K1* (Fig. 6c, d, f), potentially to cross-present viral antigen to CD8^+^ T cells, as previously shown in VSV-infection [53]. MCs are also likely responsible for this, considering they more highly express *H2-K1* (Fig. 6e, f), as well as other T cell co-stimulatory markers, and depletion of these cells additionally reduces CD8^+^ T cell proliferation (Fig. 5h). Together, this data suggests that microglia promote IFN-γ production by CD4^+^ T cells via their expression of IL-12 and/or via promoting MC maturation and APC capabilities in the CNS. Secondly, this suggests that microglia and MCs may cross-present viral antigen to CD8^+^ T cells via MHC-I, promoting their re-activation, proliferation and clearance of virus in the CNS. However, deletion of MHC-I and MHC-II on microglia or MC is required to confirm this.

### Microglia and CD8^+^ T cells are involved in viral clearance in WNE

**Fig. 7:**
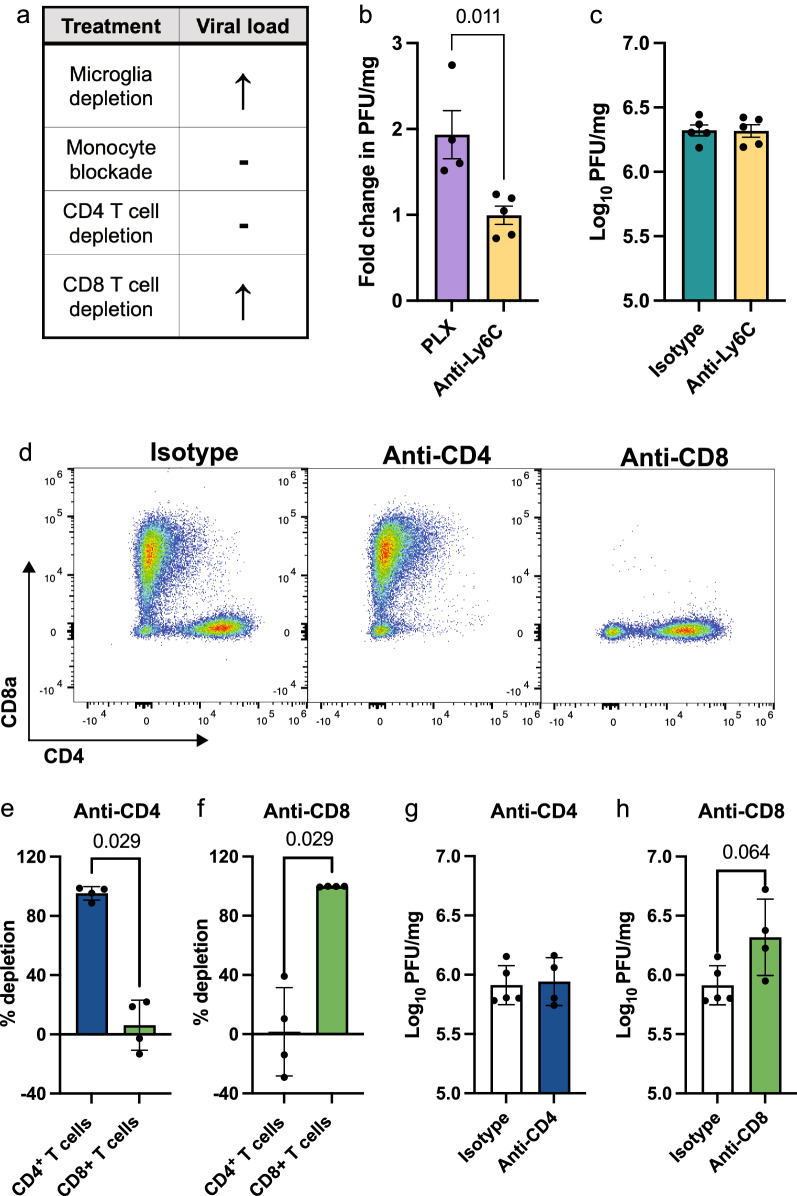
Microglia and CD8^+^ T cells are involved in viral clearance in WNV-infection **a** Summary table showing the changes in viral load with various cell subset depletions during WNV-infection. **b** Fold-change in plaque forming units (PFU) of WNV in brains from PLX5622- and anti-Ly6C-treated mice at dpi 7, relative to control fed and isotype monoclonal antibody (mAb)-treated mice, respectively, as determined by virus plaque assay. **c** PFU of WNV in brains from anti-Ly6C and isotype mAb-treated mice at dpi 7. **d** Flow cytometric plots showing the expression of CD8α and CD4 in WNV-infected mice at dpi 7 treated with either an isotype antibody, or anti-CD4 or anti-CD8 mAb. **e, f** Percent of T cell subsets depleted in mice treated with an anti-CD4 (**e**) or anti-CD8 (**f**) mAb. **g, h** PFU of WNV in brains from mice treated with anti-CD4 or an isotype control antibody (**g**) or anti-CD8 or an isotype control antibody (**h**) and sacrificed at dpi 7. Data is presented as mean ± SEM from one or two independent experiments with at least 4 mice per group

To determine the contribution of these cells to an anti-viral immune response we performed a plaque assay on brain homogenates (Fig. 7). We have previously demonstrated that PLX5622 treatment enhanced viral load in the brain [75]. However, since both microglia and MCs are reduced with this treatment, we treated mice with anti-Ly6C to determine the contribution of MCs to viral clearance. Interestingly, anti-Ly6C treatment had no effect on viral load, despite mediating a reduced T cell infiltrate (Fig. 7b, c) and being enriched for anti-viral processes (Fig. 2d, h and Additional file 5). Therefore, this data suggests that microglia have a more prominent role than MCs in viral clearance in WNV-infected mice.

To examine the contribution of T cells to viral clearance, we depleted CD4^+^ and CD8^+^ T cells in WNV-infected mice by 98% (Fig. 7d-f). CD4^+^ and CD8^+^ T cell depletion had no effect on clinical scores or the recruitment of other immune cells into the CNS (Additional file 12). CD8^+^ T cell depletion, however, increased viral titers, although this was not statistically significant (Fig. 7h), increased weight loss and reduced expression of *Ifn-*γ in the brain at dpi 7 (Additional file 12). Depletion of CD4^+^ T cells had no effect on viral load nor *Ifn-*γ expression (Fig. 7g and Additional file 12), suggesting that CD8^+^ T cells, rather than CD4^+^ T cells contribute to viral clearance in WNV-infection. Thus, the increased viral load in PLX5622-treated mice may be a result of a combination of (1) the loss of MHC-I on microglia and MCs, impeding effective CD8^+^ T cell responses, (2) the reduction in CD8^+^ T cells, (3) reduced MC maturation with microglia depletion and/or (4) reduced clearance of virus by microglia.

To investigate the interplay between microglia, MCs and CD8^+^ T cells in the CNS for viral clearance, we differentially depleted two cell types simultaneously and analysed viral load via qPCR and a virus plaque assay at WNV dpi 6 (1 day before endpoint, Additional file 13). Intriguingly, depleting both microglia and CD8^+^ T cells in the WNV-infected CNS substantially increased viral RNA, relative to mice treated with either (1) an isotype mAb, (2) anti-CD8 mAb or (3) an anti-CD8 and anti-Ly6C mAb (Additional file 13). Despite an increase in viral RNA this did not equate to a significant increase in infectious virus via a virus plaque assay. This suggests that while microglia [75] and CD8^+^ T cells (Fig. 7h) individually reduce viral load in the CNS of WNV-infected mice, cross-talk between these cells is not required to further reduce infectious virus, but may be required to reduce viral debris.

### Microglia phagocytose virus-infected neurons in WNE

**Fig. 8:**
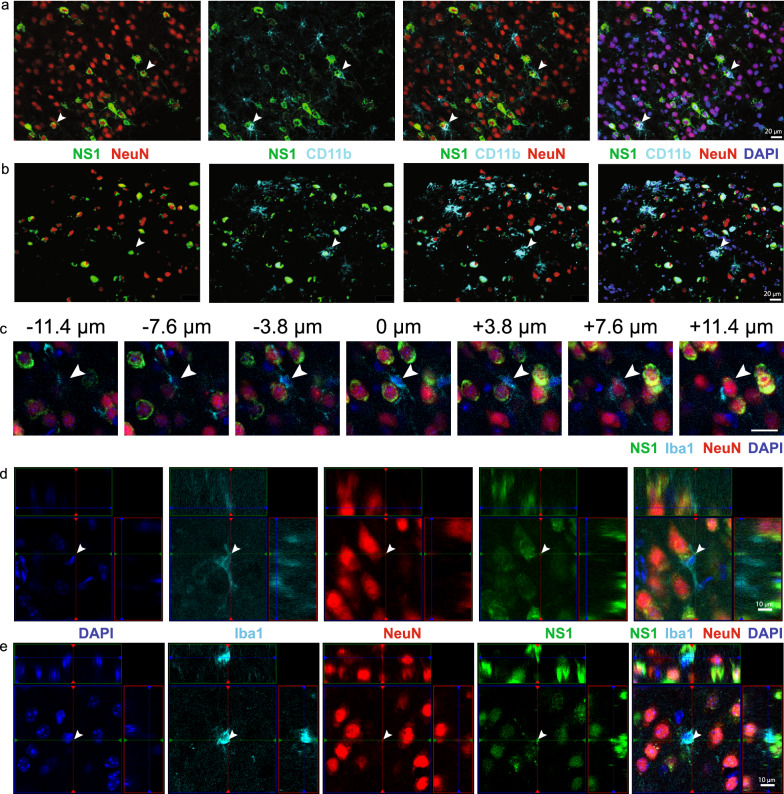
Microglia phagocytose WNV-infected neurons. **a, b** Expression of NS1 (green), NeuN (red), CD11b (cyan) and DAPI (blue) in two fields of view in brains sections from WNV-infected mice at dpi 7. Arrowheads show CD11b^+^ ramified processes (microglia) around WNV-infected neurons. **c–e** Confocal imaging of thick tissue sections stained with DAPI (blue), Iba1 (cyan), NeuN (red) and NS1 (green). **c** Image series through the z direction centred at the nucleus of the microglia (arrowhead) displaying processes wrapping around the soma**. d–e** Colocalisation of foci (arrowhead) of NeuN and NS1 inside a microglia, indicating microglia phagocytosis of an infected neuron. Scale bar represents 10 μm (**d**, **e**) or 20 μm (**a**–**c**)

Single-cell RNA analysis and depletion studies have implicated microglia in viral clearance via their expression of anti-viral genes and promotion of  T cell responses in WNE. Previous groups however, have also shown direct clearance of virus by microglia-mediated phagocytosis during CNS infection [18]. To investigate this, we examined WNV-infected brain sections via immunofluorescence. Intriguingly, we observed CD11b^+^ ramified projections around virus-infected (NS1^+^) neurons (NeuN^+^) (Fig. 8a, b). This suggests that microglia may phagocytose infected neurons and/or clear viral debris during WNE. In fact, with confocal imaging of thick tissue sections, we observed Iba1^+^ processes and cell bodies wrapping around NS1^+^ neurons and NS1^+^ particles inside Iba1^+^ cells (Fig. 8c–e). The high expression of Iba1 in combination with the ramified cell morphology indicates that these cells are microglia [76]. However, phagocytosis by MCs cannot be completely excluded. Interestingly, while microglia downregulated nominally phagocytic genes, *Cd68, Mertk* and *Trem2,* following infection, MG4 and MG5 upregulated the TAM receptor *Axl* (Fig. 3e) which can recognize apoptotic and virus-infected cells expressing phosphatidylserine, potentially enhancing viral clearance [43].

**Fig. 9:**
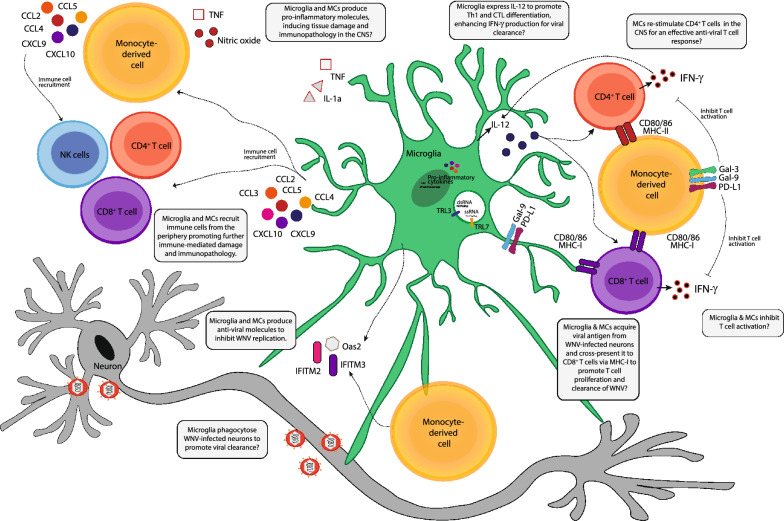
Summary figure showing the potential proinflammatory and antiviral functions of microglia and MC in WNE.

Taken together, this work suggests (1) microglia and MCs adopt unique timepoint-specific transcriptomic profiles associated with viral clearance, immune cell recruitment, antigen presentation and the production of pro-inflammatory molecules, with particular subpopulations having a more prominent role in these, (2) microglia promote CD4^+^ T cell IFN-γ production, potentially via their expression of IL-12 or via promoting MC maturation in the CNS, (3) microglia and/or MCs promote CD8^+^ T cell proliferation for viral clearance, potentially via cross presentation, (4) microglia and MCs express T cell inhibitory markers to reduce T cell activity and (5) microglia phagocytose virus-infected cells (Fig. 9).

## Discussion

Microglia have been implicated in promoting both disease resolution and progression in the acute and post-infectious phase of viral infection. However, the limited tools that specifically target microglia have impeded our ability to elucidate their precise contributions to pathology, with many of these studies relying on microglia-depleting agents, primarily PLX5622, which cause off-target effects, both in the CNS and periphery [32, 41, 42, 75]. Defining anti-viral microglial response mechanisms is critically required to identify targets for immune intervention. Using a combination of experimental approaches, we have defined the shared and differential contributions of resident and peripherally-derived myeloid cells in the virus-infected CNS.

Single-cell transcriptomic analysis on sorted myeloid cells from murine brains in the lethal progression of WNE demonstrated 6 microglia and 3 MC clusters. Over the course of infection, microglia clusters formed two distinct lineages via trajectory analysis on scRNA-seq data, specifically a functionally adaptive and a stressed/dying response. The functionally adaptive microglial clusters changed from homeostatic populations, MG1 and MG2, enriched for CNS regulatory processes, into MG4, demonstrating CNS regulatory and anti-viral functions, and then into MG5, highly expressing chemotactic molecules for immune cell recruitment by dpi 7. Intriguingly, cytometric analysis also demonstrated timepoint-specific microglia alterations, with microglia in the mock-infected, dpi 5 and dpi 7 mice showing distinct protein phenotypes by progressively upregulating CD45 and CD64 and downregulating F4/80, CX3CR1, TMEM119 [76].

Supporting previous work, we have demonstrated neuroprotective roles for microglia in CNS infection [7, 13, 18, 53, 66–68, 70, 79, 88, 92, 99, 103], including promoting T cell effector functions [7, 19, 49, 53, 67, 99, 103] and viral clearance via the expression of ISGs and the phagocytosis of viral antigen [17, 18]. However, we also show that microglia may play a neurotoxic role by expressing pro-inflammatory genes, *Tnf, Il1a* and chemokines, *Ccl2, Ccl2-5, Cxcl9* and *Cxcl10* inducing the recruitment of inflammatory MCs and T cells, likely contributing to immunopathology and neuroinflammation [22–25, 101]. Ironically, this is in line with earlier work using less specific microglia manipulation tools, including in minocycline-treated *ex-vivo* spinal cord slice culture [60] and *Peli1*^–/–^ mice [47], which demonstrated reduced neuroinflammation paralleled by reduced microglial activation. This therefore suggests that PLX5622 microglia depletion in WNE is not only neuroprotective because of the reduction in NO-producing Ly6C^hi^ inflammatory MCs [23–25, 75], but also due to the depletion of pathogenic microglia [74]. While cell-specific depletion and gene deletion models and/or in vivo fate-mapping is required to confirm these findings, this work nevertheless identifies potential targets for therapeutic intervention to suppress damaging chemokine responses in microglia.

In contrast to microglia, MCs formed 3 distinct clusters, with MC1 present predominantly at dpi 5 and MC2 and MC3 at dpi 5 and 7, respectively. These distinct phenotypes may represent a trajectory of maturation from MC1 to MC3 with a single BM-derived phenotype infiltrating the CNS or they may immigrate into the CNS as unique subsets pre-programmed in the BM. Alternatively, they may arise due to CNS entry at different anatomical locations or exposure to different brain regions with different infection rates and cytokine environments [72, 77, 78]. However, we cannot exclude the possibility that a small number of these cells represent other CNS-resident border-associated macrophages. While the origins of these phenotypes require further investigation, previous work has demonstrated a pathological role for MCs in WNE, with experimental manipulation designed to reduce their infiltration ameliorating the disease phenotype and/or promoting survival [23–25, 75, 76, 83]. Adding to this body of work, more detailed transcriptomic analysis suggests that these cells are also neuroprotective, expressing anti-viral ISGs modulating WNV replication, including *Ifitm2, Ifitm3* and *Oas2* [5, 28, 34]. These cells were also enriched for apoptotic cell clearance (MC2 and MC3) and wound healing (MC2). However, MC2 and MC3 differentially expressed genes associated with reactive oxygen species production, including *Cybb* and *Nos2,* respectively, and were associated with apoptosis and production of TNF and IL-1β. Thus, while they may promote viral clearance, the sheer number of these cells infiltrating the CNS, some 2.5 million MCs by dpi 7, with their infiltration further promoting monocyte chemotaxis, likely contributes to the severe immunopathology observed.

Treating mice with PLX5622, anti-Ly6C mAb or clodronate to deplete microglia and/or MCs in the CNS reduced the recruitment of NK and T cells into the virus-infected brain, supporting a role for resident and infiltrating myeloid cells in CNS trafficking. While we cannot exclude the possibility of off-target effects produced by clodronate and anti-Ly6C mAb on peripheral NK and T cells, resulting in their reduced infiltration into the CNS, the scRNA-seq data shows expression of relevant chemotactic genes by microglia and MCs, including *Ccl5, Cxcl9, Cxcl10* and *Cxcl16.* These are involved in immune cell recruitment in CNS virus infection, making it likely that recruitment to the CNS is largely driven by microglia and MCs [26, 38, 52, 63, 82, 108, 110]. Nevertheless, since peripheral immune cell recruitment was not completely abolished in microglia- and/or MC-depleted mice, this suggests that other resident CNS cells, such as astrocytes and neurons, may also contribute to this. Thus, targeting the expression of these molecules in both resident and infiltrating populations may provide a therapeutic target for immune intervention to reduce the neuroinflammatory response.

We also suggest a shared role for microglia and MCs in viral clearance, via their expression of anti-viral ISGs including *Oas2, Ifitm2 and Ifitm3.* Additionally, we showed microglial ramified processes wrapping around virus-infected neurons, suggesting direct clearance of virus by microglia. The use of cell-specific histological markers, however, are required to exclude the possibility of MC-mediated phagocytosis of virus-infected neurons. Nonetheless, it has previously been demonstrated that microglia phagocytose virus in human tissue infected with WNV [56], in an *ex-vivo* slice culture model of WNV-infection [59], as well as in PRV-infection, where P2RY12 was required for microglia-mediated phagocytosis of viral debris, which prevented the lethal spread of virus in the CNS [18]. However, depletion of either microglia or MCs in the CNS only modestly increased or had no effect viral load. Perhaps in our model of WNV-infection the rate of infection outstrips the rate of virus clearance. Irrespective, clearly the infiltration of MCs into the CNS induces damage, likely preventing effective viral clearance. Mechanisms that dampen microglia- and MC-mediated inflammation in the CNS and promote their anti-viral functions, may thus aid in therapeutic intervention.

Type I IFNs such as IFN-α and IFN-β are required to control viral replication and spread to the periphery and CNS during WNV infection [65]. In our study scRNA-seq data demonstrated that neither microglia nor MC clusters expressed *Ifna1* (Additional file 6). While microglia and MCs may produce other *Ifna* subtypes not examined here, this work strongly suggests that *Ifna1* is principally produced by other cells in WNE, presumably WNV-infected neurons. This may not be surprising, as there is little evidence for infection of any cells besides neurons in WNE. Other studies suggest that microglia are the main source of type I IFNs in CNS infection, however, these include more pleootropic viruses, such as HSV, which also infect microglia [61]. Indeed, in neurotropic VSV infection, IFNAR signaling in astrocytes and neurons regulated microglial activation, proliferation, and recruitment to sites of infection which was required to form an “innate immune barrier”, preventing the lethal spread of virus [13]. Similarly, neuronally-produced *Ifna1* may be required for microglial and MC interferon-signaling and anti-viral responses in WNE – potentially more so in MCs, since MCs and not microglia expressed its cognate receptor, *Ifnar1* during infection (Additional file 6).

Over the course of WNE, microglia upregulated MHC-I/H2-K^b^. This is presumably a response to local soluble factors, including interferons, rather than a direct virus effect, as these cells are not infected by WNV [36, 46]. Enhanced expression of MHC-I may enable microglia to cross-present antigen acquired from neurons to CD8^+^ T cells to promote activation and virus clearance [27, 53]. The reduction in CD8^+^ T cell proliferation at WNV dpi 5 in the absence of microglia also supports a role for microglia in cross-presentation and CD8^+^ T activation. Alternatively, microglia and/or just MCs may be responsible for this, as anti-Ly6C-treated mice also demonstrate reduced CD8^+^ T cell proliferation. More specific depletion tools are required to elucidate this in more detail.

Intriguingly, microglia did not upregulate MHC-II, commonly argued to be an activation/proinflammatory marker in infection [16], despite the presence of IFN-γ during WNE. However, its upregulation may be context- and virus-specific, as a study using neurotropic VSV also demonstrated only a modest increase in MHC-II, relative to MHC-I [53], while in JHMV infection microglia upregulated MHC-II transcripts [82]. We demonstrated a decreased expression of IFN-γ by CD4^+^ T cells in PLX5622-treated mice, suggesting CD4^+^ T cell impairment is due to the absence of microglia, rather than absence of  microglial MHC-II expression. Instead, reduced T cell responses may be explained by the loss of IL-12-expressing microglia, a molecule which promotes Th1 differentiation. Alternatively, since MCs were more highly enriched for MHC-II molecules and processes, compared to microglia, microglia may be required indirectly for co-stimulation and T cell activation capabilities, via promoting MC maturation in the CNS. However, recent studies demonstrate that PLX5622 affects naïve T cell development and reduces peripheral antigen-presenting molecules [19, 42], suggesting that PLX5622 impairs priming of peripheral T cells. Therefore, the reduced expression of IFN-γ by CD4^+^ T cells may be an off-target effect of PLX5622 in our study. More specific microglia depletion methods are required to confirm these results.

This study advocates caution in the use of cell-depletion models alone to elucidate the precise contributions of specific cells to normal physiological immune responses. Secondly, it emphasizes the complexity of myeloid responses to perturbation, with microglia and MCs each adopting unique transcriptional profiles that adapt with disease progression. Thirdly, we show that while microglia and MC have anti-viral functions that may be neuroprotective, these cells also simultaneously produce pro-inflammatory mediators that likely contribute to an overexuberant inflammatory response. This strongly challenges historical dogma surrounding the binary M1 (pro-inflammatory) vs M2 (anti-inflammatory) myeloid phenotypes. We further demonstrate a role for both of these cells in immune cell recruitment, T cell responses and viral clearance. This study highlights potential targets for effective immune intervention that could reduce damaging inflammatory myeloid responses in the CNS without impairing viral clearance or crucial neuroprotective mechanisms.

## Supplementary Information

**Additional file 1.** Gating strategy used to sort microglia and MCs from mock- and WNV-infected mice for scRNA-seq analysis. Circulating monocytes were not identified, however, the presence of these cells is expected to be low in the brain due to transcardiac perfusion. Single-cell brain suspensions were sorted on the 10-laser Influx cell sorter using the FACSDiva Programme. Shown in the figure is a WNV-infected brain at dpi 7. H&Es of sorted microglia and MCs populations are shown with their respective gates.

**Additional file 2.** Single-cell RNA gene list and gene modules determined by Monocle3.

**Additional file 3.** Trajectory analysis with Monocle3 and Slingshot. **a** Dot plot showing the expression of nominal microglia and MCs genes in microglia and MC clusters. **b**, **c** UMAP plots coloured by pseudotime, determined using trajectory analysis with Monocle3 on both microglia and MC clusters. MG1 (**b**) or MC1 (**c**) were used as the starting cells. Increased pseudotime indicates further distance from starting cells (i.e., MG1 and MC1). **d**, **e** UMAP plots showing two lineage trajectories on microglia clusters using Slingshot. MG1 was used as the starting cell cluster.

**Additional file 4.** Top markers per scRNA-seq cluster.

**Additional file 5.** Top gene ontology processes associated with differentially- expressed genes.

**Additional file 6.** Expression of select genes in scRNA-seq microglia and MC clusters.

**Additional file 7.** Genes up- and down-regulated by scRNA-seq microglia clusters.

**Additional file 8.** Gene ontology processes associated with genes downregulated by microglia.

**Additional file 9.** Gene ontology processes associated with genes upregulated by microglia.

**Additional file 10.** Expression of select genes in whole brains of mock- and WNV-infected mice treated with PLX5622 or anti-Ly6C. Gene expression was determined by qPCR and normalised to the housekeeping gene, *Rpl13a*. Mice were fed PLX5622 for 21 days prior to infection and until dpi 5 or 7. Anti-Ly6C was injected on dpi 5 and 6. Data is presented as mean ± SEM from 1-3 independent experiments with at least 4 mice per group.

**Additional file 11.** PLX5622 treatment does not affect peripheral T cell proliferation. **a**–**f** Number (**a**–**c**) and frequency (**d**–**f**) of BrdU^+^ proliferating T cells in mock-infected vs WNV dpi 7 mice (**a**, **d**), mock-infected, *Ctrl *vs *PLX* mice (**b**, **e**) and WNV dpi 7, *Ctrl *vs *PLX* mice (**c**, **f**).  Mice were fed PLX5622 for 21 days prior to infection and until dpi 7. Data is presented as mean ± SEM from one or two independent experiments with at least 5 mice per group.

**Additional file 12.** T cell depletion in WNV-infected mice. **a**, **b** Disease score (**a**) and percent weight loss at dpi 7 (**b**) of mice treated with an isotype control, anti-CD4 or anti-CD8 monoclonal antibody (mAb). **c** Expression of *Ifn-γ*, as determined by qPCR in the brain from mice treated with an isotype control, anti-CD4 or anti-CD8 mAb. **d**, **e** Number of cells in the brain of WNV-infected mice treated with an isotype control, anti-CD4 (**d**) or anti-CD8 mAb (**e**). Mice were treated with mAbs at dpi 4 and 6. Data is presented as mean ± SEM from one or two independent experiments with at least 4 mice per group.

**Additional file 13.** Microglia and CD8^+^ T cell cross-talk is not required for viral clearance in WNV-infected mice. **a**–**c** Number of CD8^+^ T cells (**a**), Ly6C^hi^ MCs (**b**) and microglia (**c**) in the brains of WNV-infected mice at dpi 6 treated with an isotype control monoclonal antibody (mAb), anti-CD8 mAb alone, anti-CD8 and anti-Ly6C mAb or anti-CD8 mAb and PLX5622. **d**–**f** Percent weight loss at dpi 6 (**d**), expression of *Wnv* as determined by qPCR (**e**) and virus plaque assay for the quantification of infectious virus in brains from WNV-infected mice at dpi 6 (**f**) and treated with an isotype control mAb, anti-CD8 mAb alone, anti-CD8 and anti-Ly6C mAb, or anti-CD8 mAb and PLX5622. Mice were fed PLX5622 for 21 days prior to infection and until dpi 6, while mAbs were administered on dpi 4 and 5. Data is presented as mean ± SEM from one independent experiments with at least 3 mice per group.

## Data Availability

The datasets used and/or analysed during the current study are available from the corresponding author on reasonable request.
