# Supplementary figures and images for: Temporal tracking of microglial and monocyte single-cell transcriptomics in lethal flavivirus infection
Source: Acta Neuropathol Commun. 2023 Apr 4;11:60. doi: 10.1186/s40478-023-01547-4 (PMC10074823; doi:10.1186/s40478-023-01547-4)

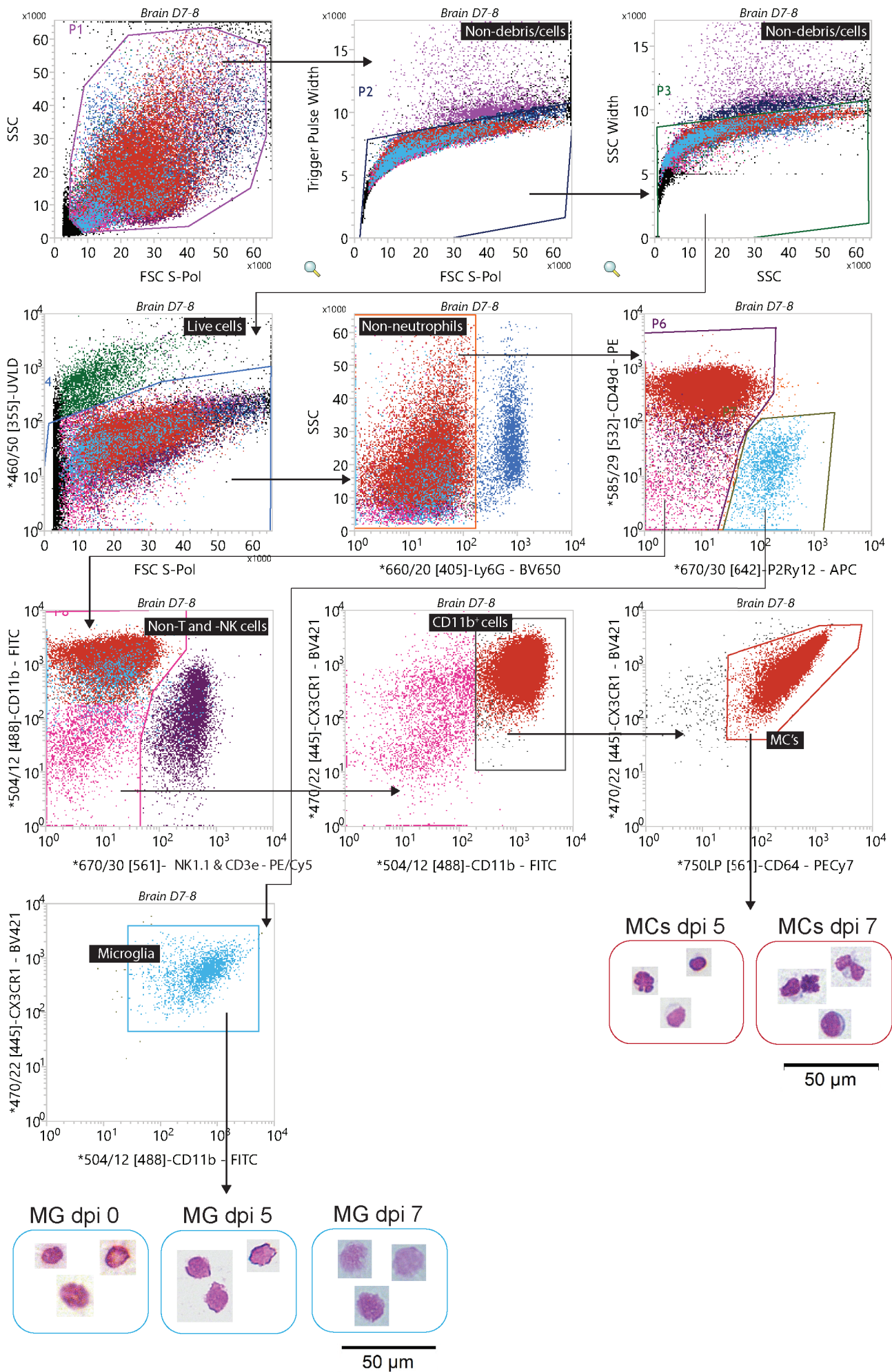

Supplement: Supplementary file 1 — Additional file 1. Gating strategy used to sort microglia and MCs from mock- and WNV-infected mice for scRNA-seq analysis. Circulating monocytes were not identified, however, the presence of these cells is expected to be low in the brain due to transcardiac perfusion. Single-cell brain suspensions were sorted on the 10-laser Influx cell sorter using the FACSDiva Programme. Shown in the figure is a WNV-infected brain at dpi 7. H&Es of sorted microglia and MCs populations are shown with their respective gates. [file 40478_2023_1547_MOESM1_ESM.pdf]

**a Microglia & MC markers**

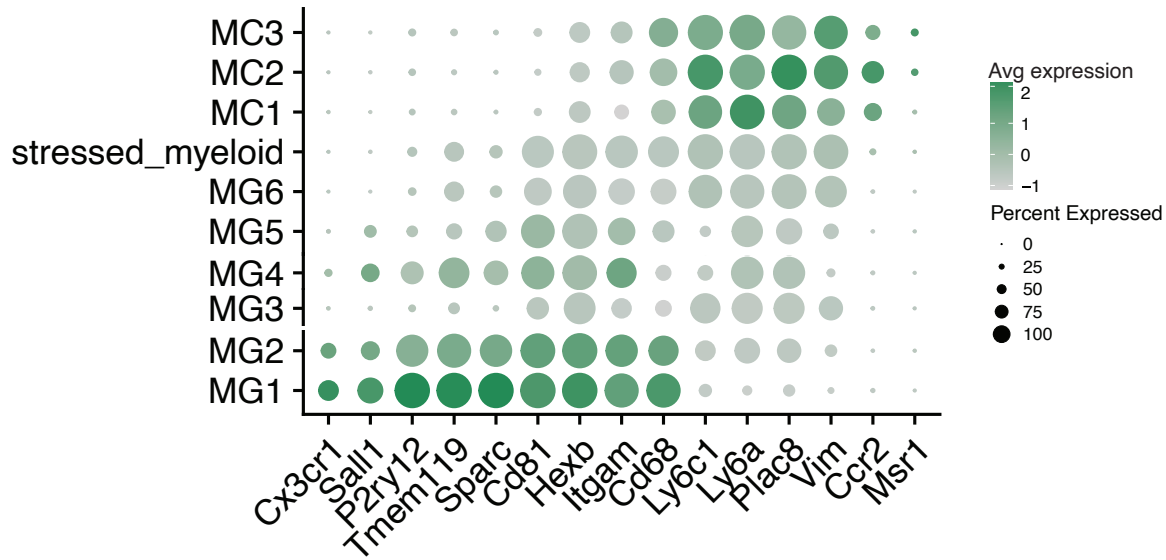

**Trajectory analysis**

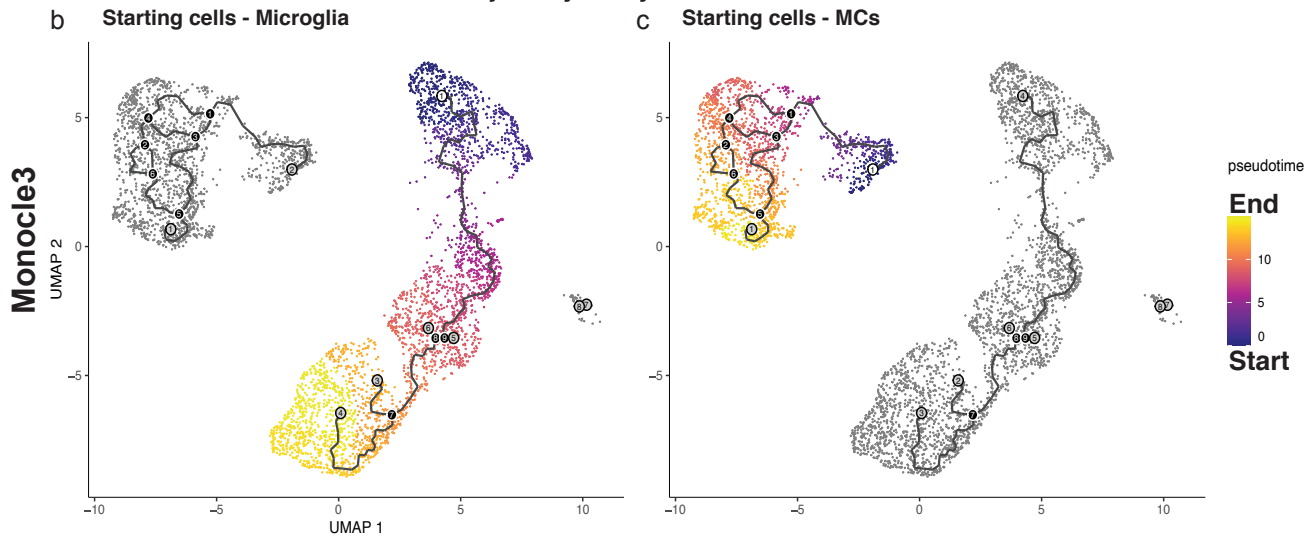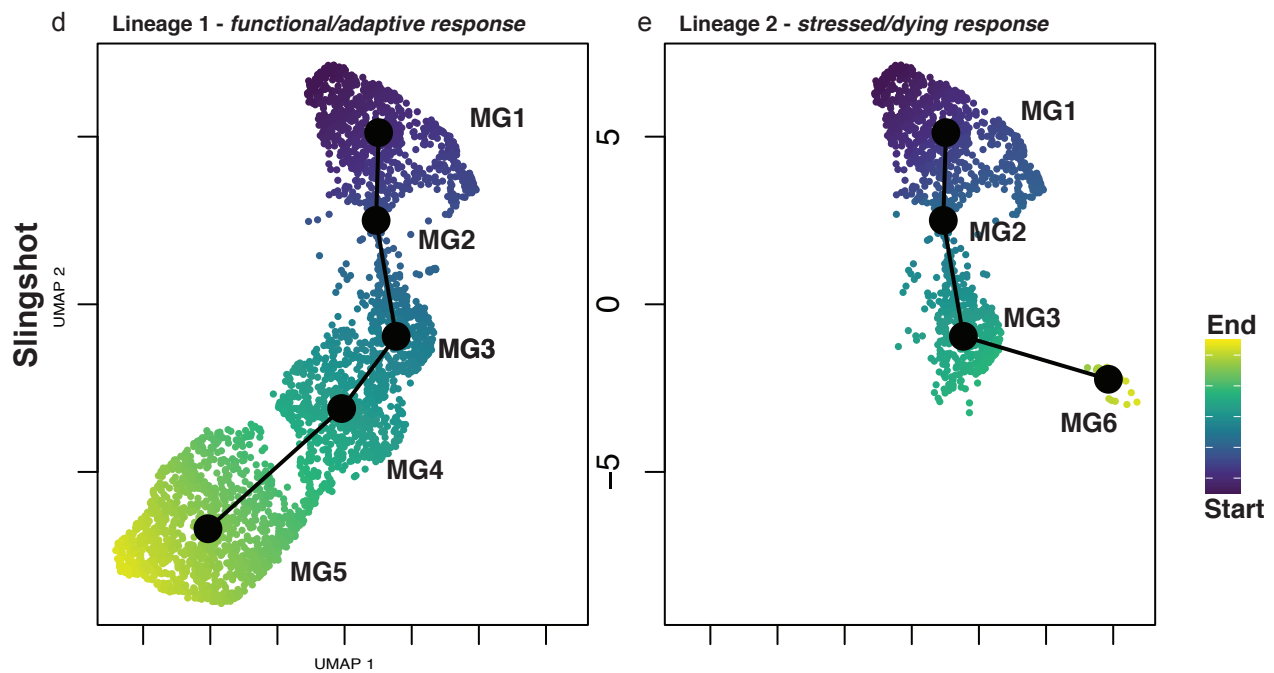

Supplement: Supplementary file 3 — Additional file 3. Trajectory analysis with Monocle3 and Slingshot. a Dot plot showing the expression of nominal microglia and MCs genes in microglia and MC clusters. b, c UMAP plots coloured by pseudotime, determined using trajectory analysis with Monocle3 on both microglia and MC clusters. MG1 (b) or MC1 (c) were used as the starting cells. Increased pseudotime indicates further distance from starting cells (i.e., MG1 and MC1). d, e UMAP plots showing two lineage trajectories on microglia clusters using Slingshot. MG1 was used as the starting cell cluster. [file 40478_2023_1547_MOESM3_ESM.pdf]

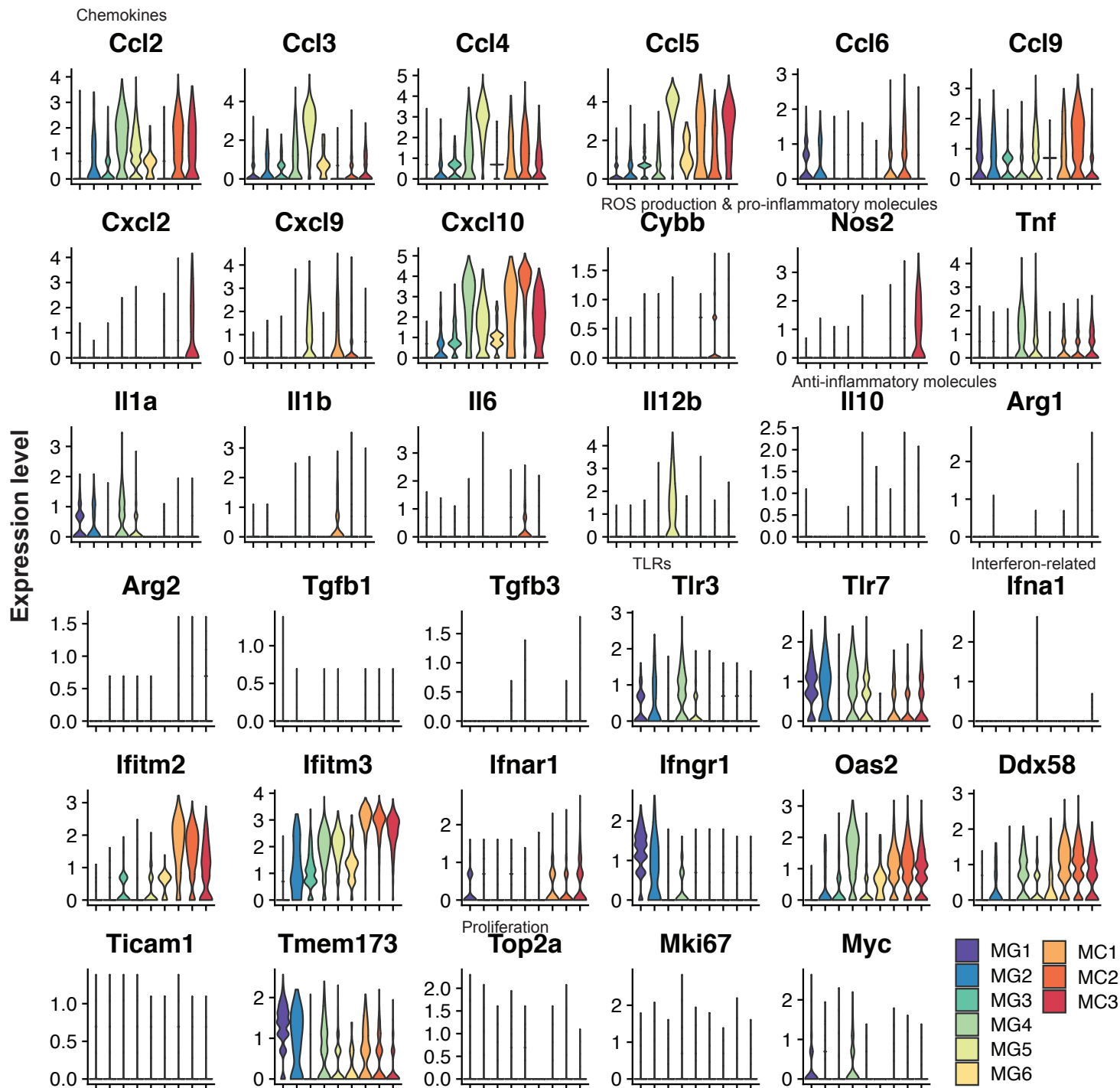

Supplement: Supplementary file 6 — Additional file 6. Expression of select genes in scRNA-seq microglia and MC clusters. [file 40478_2023_1547_MOESM6_ESM.pdf]

Mock vs dpi 7

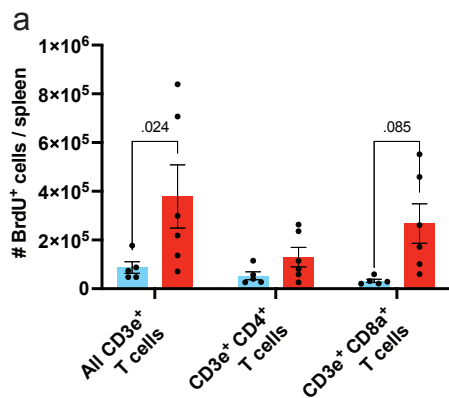

Mock: *Ctrl* vs *PLX*

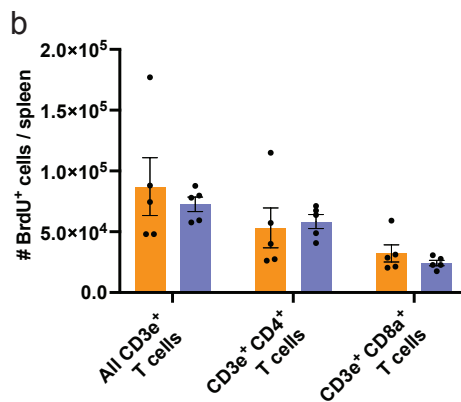

dpi7: *Ctrl* vs *PLX*

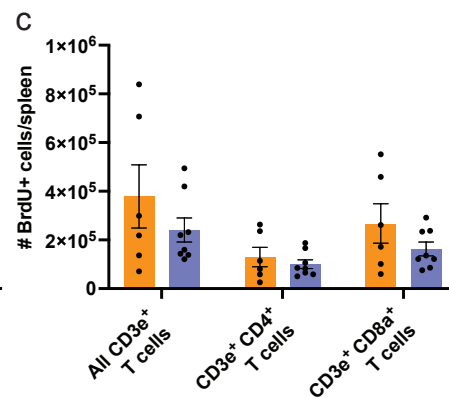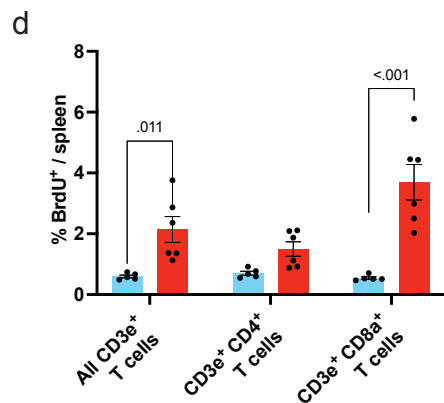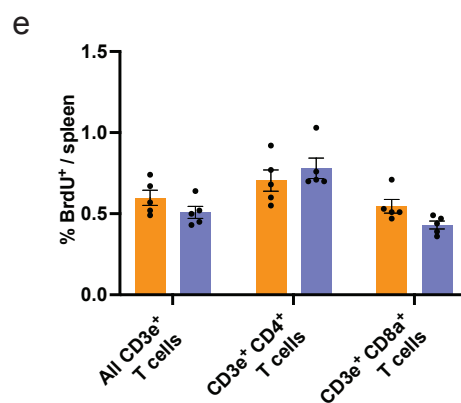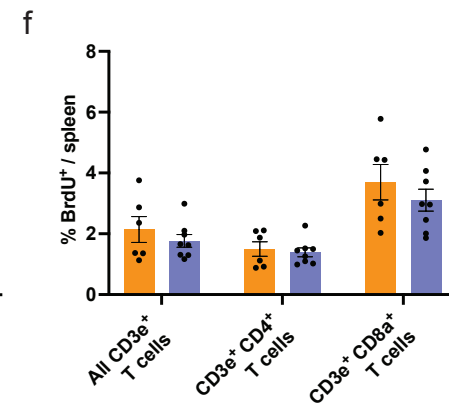

Mock dpi 7

Ctrl PLX

Supplement: Supplementary file 11 — Additional file 11. PLX5622 treatment does not affect peripheral T cell proliferation. a–f Number (a–c) and frequency (d–f) of BrdU+ proliferating T cells in mock-infected vs WNV dpi 7 mice (a, d), mock-infected, Ctrl vs PLX mice (b, e) and WNV dpi 7, Ctrl vs PLX mice (c, f). Mice were fed PLX5622 for 21 days prior to infection and until dpi 7. Data is presented as mean ± SEM from one or two independent experiments with at least 5 mice per group. [file 40478_2023_1547_MOESM11_ESM.pdf]

a Disease score @ dpi 7

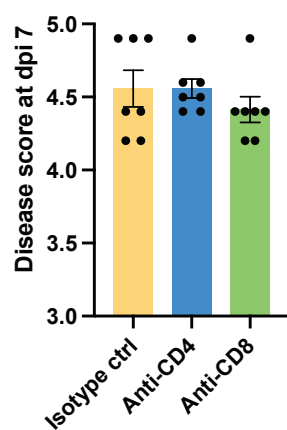

b % weight loss @ dpi 7

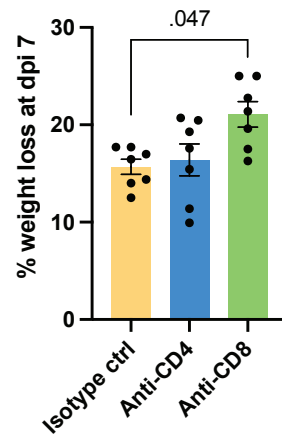

c *Ifn-γ*

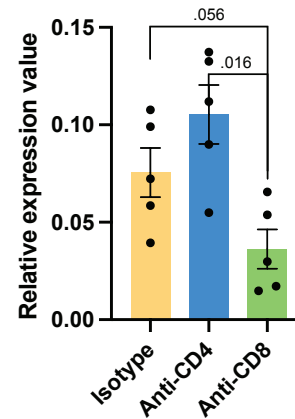

d Anti-CD4

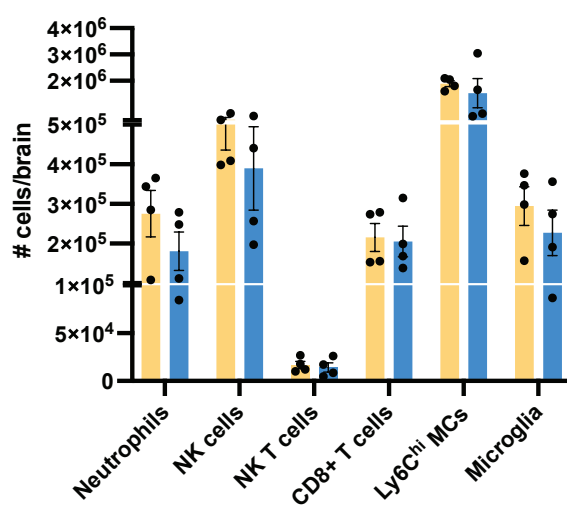

e Anti-CD8

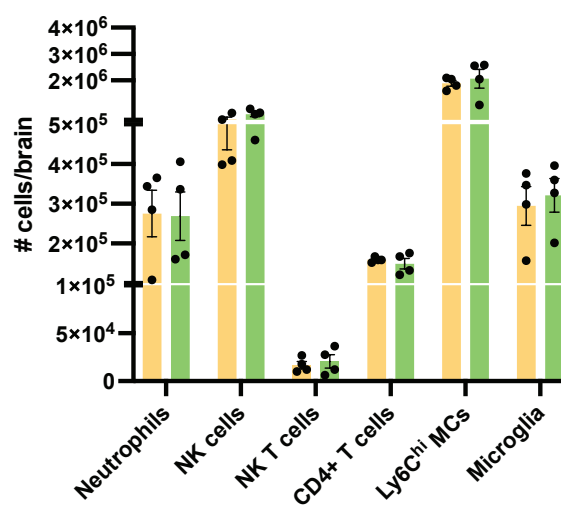

Isotype ctrl Anti-CD4 Anti-CD8

Supplement: Supplementary file 12 — Additional file 12. T cell depletion in WNV-infected mice. a, b Disease score (a) and percent weight loss at dpi 7 (b) of mice treated with an isotype control, anti-CD4 or anti-CD8 monoclonal antibody (mAb). c Expression of Ifn-γ, as determined by qPCR in the brain from mice treated with an isotype control, anti-CD4 or anti-CD8 mAb. d, e Number of cells in the brain of WNV-infected mice treated with an isotype control, anti-CD4 (d) or anti-CD8 mAb (e). Mice were treated with mAbs at dpi 4 and 6. Data is presented as mean ± SEM from one or two independent experiments with at least 4 mice per group. [file 40478_2023_1547_MOESM12_ESM.pdf]

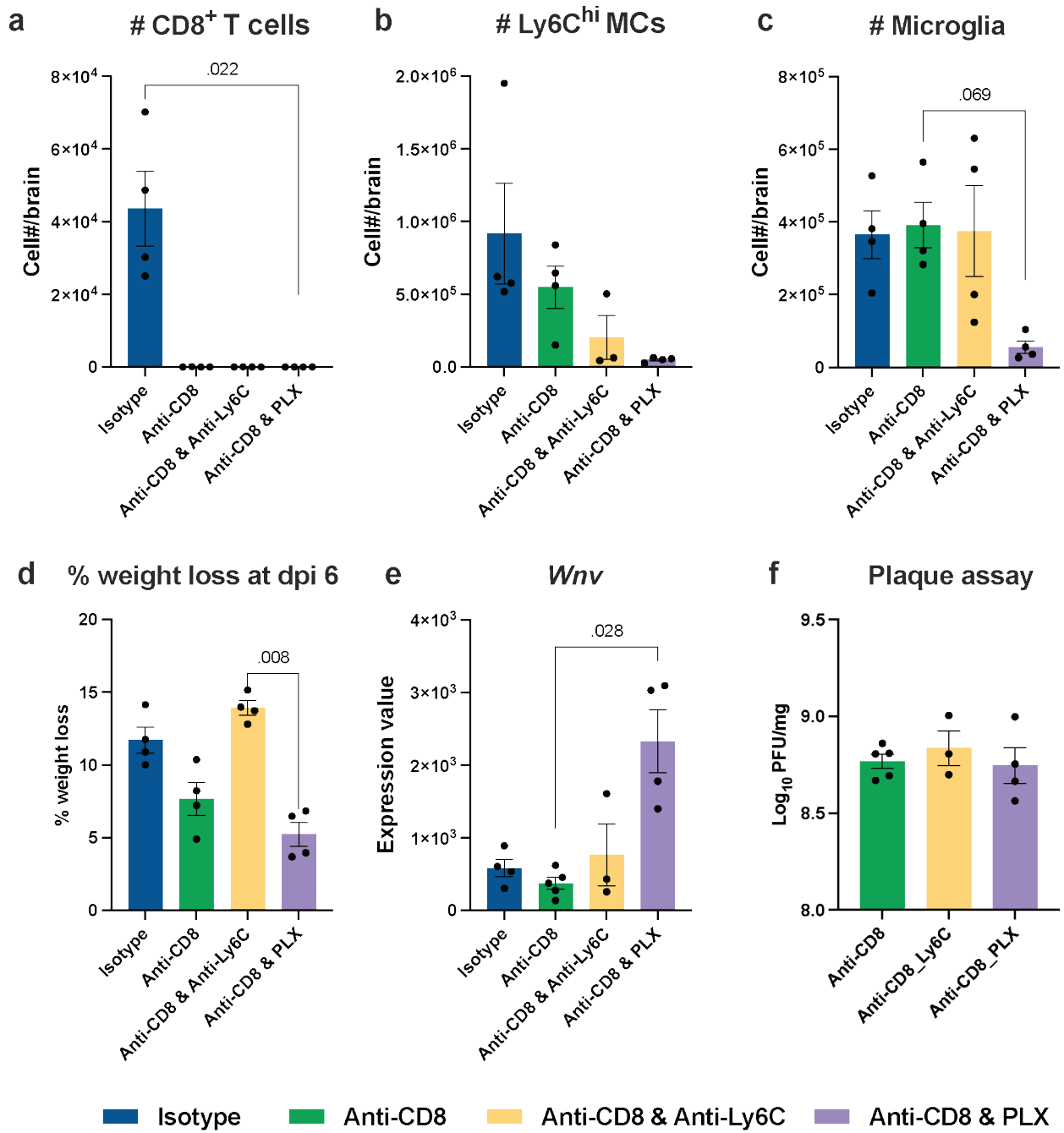

Supplement: Supplementary file 13 — Additional file 13. Microglia and CD8+ T cell cross-talk is not required for viral clearance in WNV-infected mice. a–c Number of CD8+ T cells (a), Ly6Chi MCs (b) and microglia (c) in the brains of WNV-infected mice at dpi 6 treated with an isotype control monoclonal antibody (mAb), anti-CD8 mAb alone, anti-CD8 and anti-Ly6C mAb or anti-CD8 mAb and PLX5622. d–f Percent weight loss at dpi 6 (d), expression of Wnv as determined by qPCR (e) and virus plaque assay for the quantification of infectious virus in brains from WNV-infected mice at dpi 6 (f) and treated with an isotype control mAb, anti-CD8 mAb alone, anti-CD8 and anti-Ly6C mAb, or anti-CD8 mAb and PLX5622. Mice were fed PLX5622 for 21 days prior to infection and until dpi 6, while mAbs were administered on dpi 4 and 5. Data is presented as mean ± SEM from one independent experiments with at least 3 mice per group. [file 40478_2023_1547_MOESM13_ESM.pdf]
