# Supplementary material for: Temporal tracking of microglial and monocyte single-cell transcriptomics in lethal flavivirus infection
Source: Acta Neuropathol Commun. 2023 Apr 4;11:60. doi: 10.1186/s40478-023-01547-4 (PMC10074823; doi:10.1186/s40478-023-01547-4)
Supplement: Supplementary file 10 — Additional file 10. Expression of select genes in whole brains of mock- and WNV-infected mice treated with PLX5622 or anti-Ly6C. Gene expression was determined by qPCR and normalised to the housekeeping gene, Rpl13a. Mice were fed PLX5622 for 21 days prior to infection and until dpi 5 or 7. Anti-Ly6C was injected on dpi 5 and 6. Data is presented as mean ± SEM from 1-3 independent experiments with at least 4 mice per group. [file 40478_2023_1547_MOESM10_ESM.pdf]

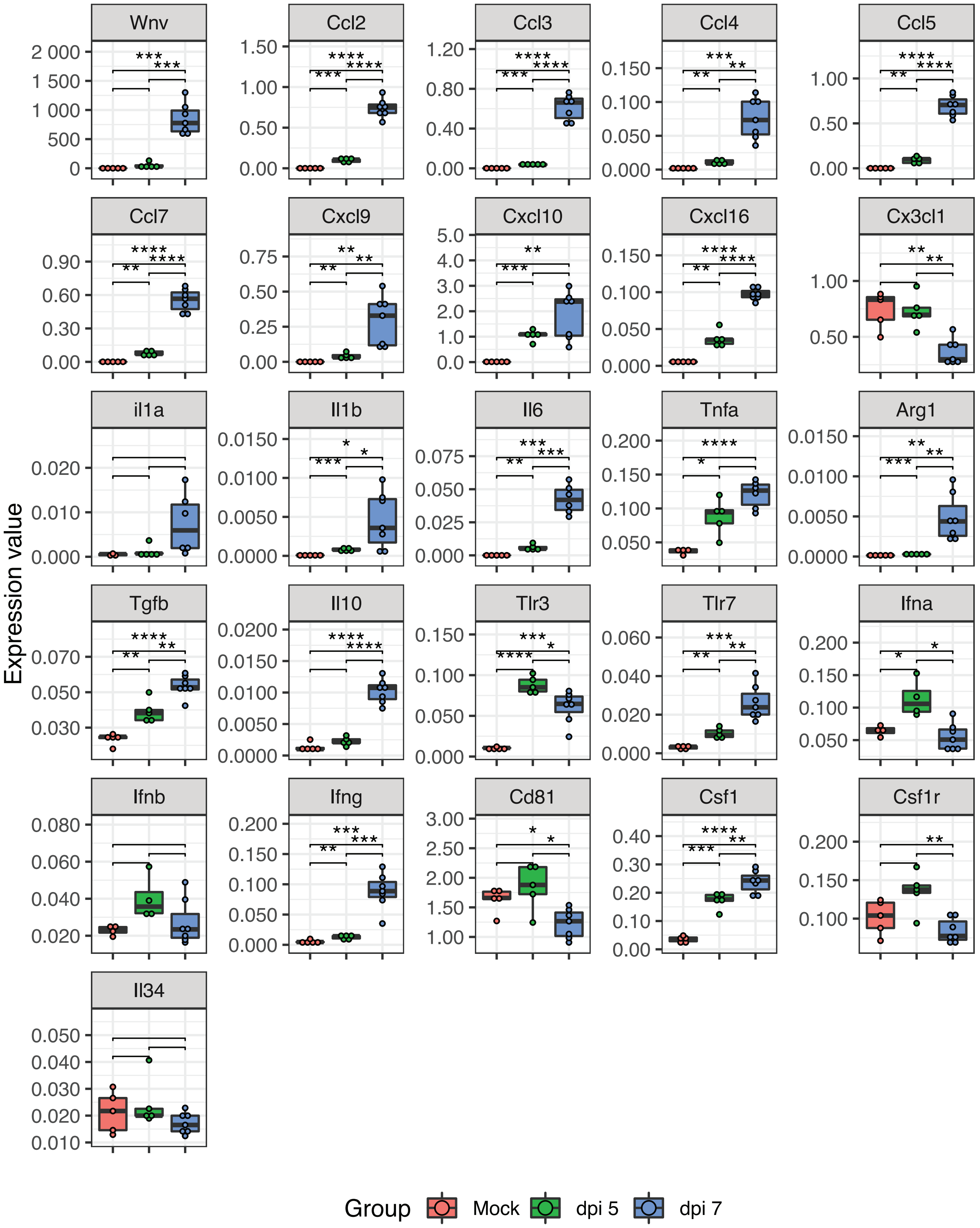

Wnv
Ccl2
Ccl3
Ccl4
Ccl5
Ccl7
Cxcl9
Cxcl10
Cxcl16
Cx3cl1
il1a
Il1b
Il6
Tnfa
Arg1
Tgfb
Il10
Tlr3
Tlr7
Ifna
Ifnb
Ifng
Cd81
Csf1
Csf1r
Il34
Expression value
Group
Mock
dpi 5
dpi 7

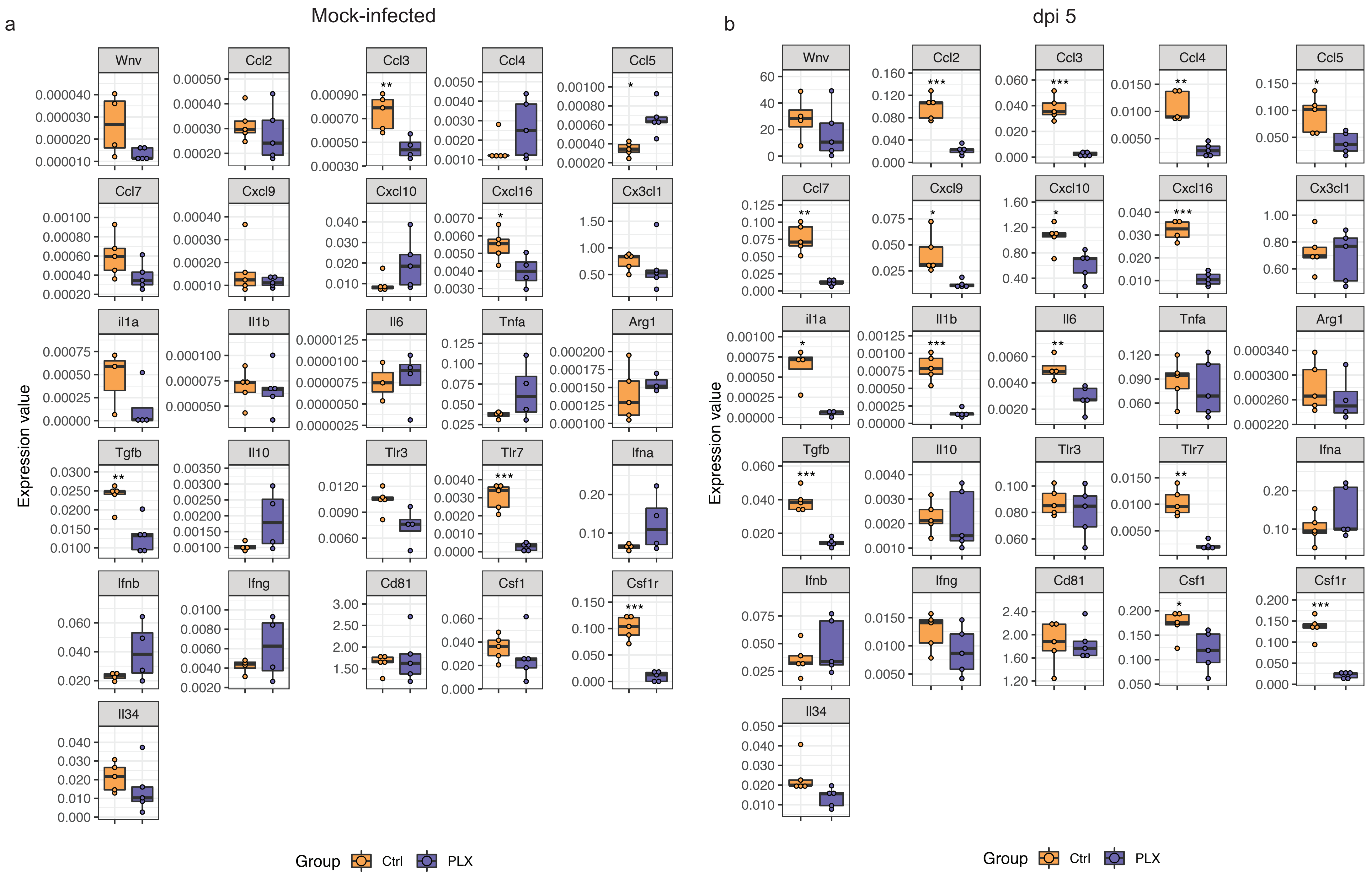
a
Mock-infected
b
dpi 5
Expression value
Wnv
Ccl2
Ccl3
Ccl4
Ccl5
Ccl7
Cxcl9
Cxcl10
Cxcl16
Cx3cl1
il1a
Il1b
Il6
Tnfa
Arg1
Tgfb
Il10
Tlr3
Tlr7
Ifna
Ifnb
Ifng
Cd81
Csf1
Csf1r
Il34
Group
Ctrl
PLX

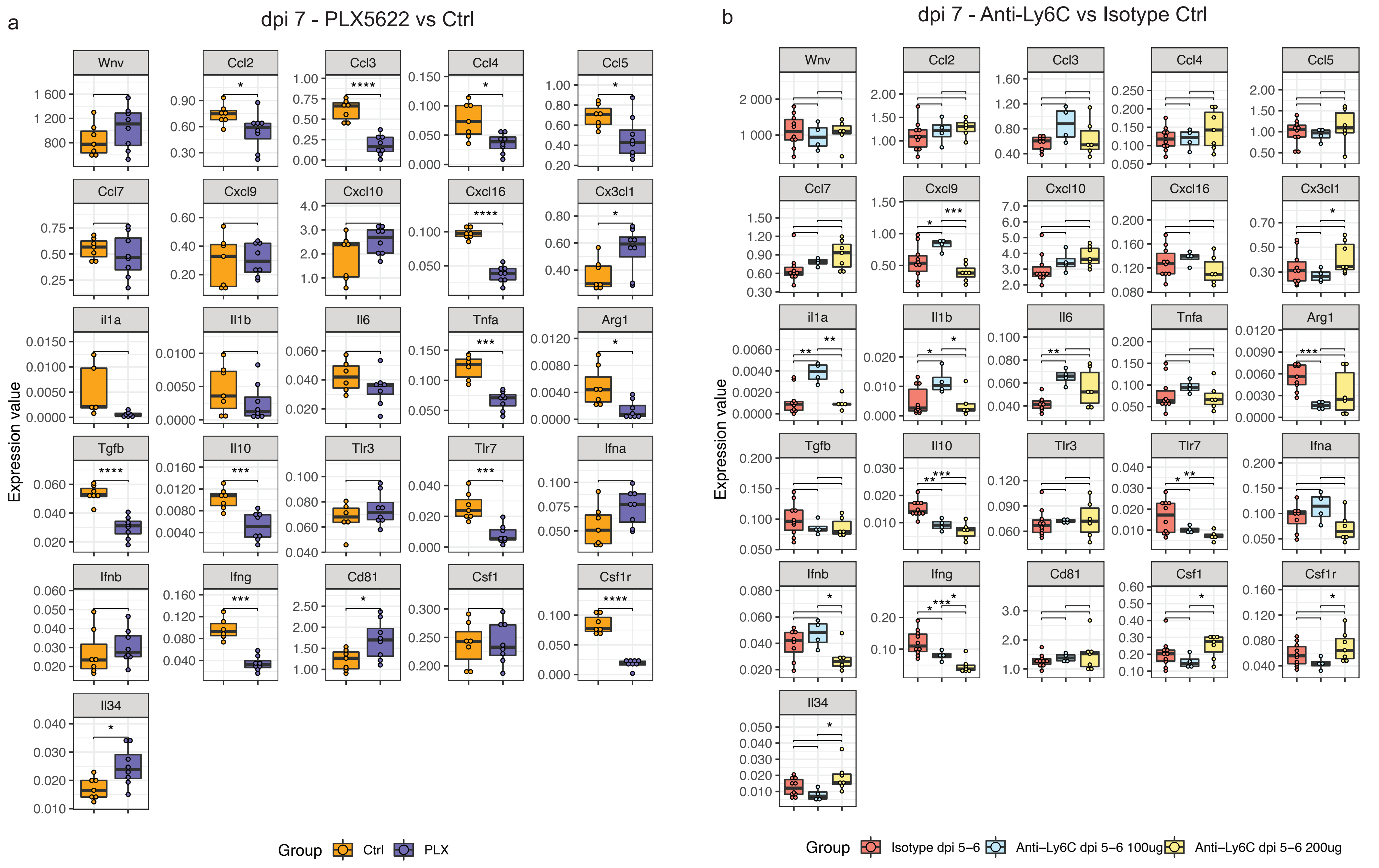
a
dpi 7 - PLX5622 vs Ctrl
Wnv
Ccl2
Ccl3
Ccl4
Ccl5
Ccl7
Cxcl9
Cxcl10
Cxcl16
Cx3cl1
il1a
Il1b
Il6
Tnfa
Arg1
Tgfb
Il10
Tlr3
Tlr7
Ifna
Ifnb
Ifng
Cd81
Csf1
Csf1r
Il34
Expression value
Group
Ctrl
PLX
b
dpi 7 - Anti-Ly6C vs Isotype Ctrl
Wnv
Ccl2
Ccl3
Ccl4
Ccl5
Ccl7
Cxcl9
Cxcl10
Cxcl16
Cx3cl1
il1a
Il1b
Il6
Tnfa
Arg1
Tgfb
Il10
Tlr3
Tlr7
Ifna
Ifnb
Ifng
Cd81
Csf1
Csf1r
Il34
Expression value
Group
Isotype dpi 5–6
Anti–Ly6C dpi 5–6 100ug
Anti–Ly6C dpi 5–6 200ug
